# SUMO Conjugation to BZR1 Enables Brassinosteroid Signaling to Integrate Environmental Cues to Shape Plant Growth

**DOI:** 10.1016/j.cub.2021.01.060

**Published:** 2021-02-08

**Authors:** Moumita Srivastava, Anjil K. Srivastava, Beatriz Orosa-Puente, Alberto Campanaro, Cunjin Zhang, Ari Sadanandom

(Current Biology *30*, 1410–1423.e1–e3; April 20, 2020)

In Figure 4D of this article, the label of y axis should be *BRZ/MS,* not *MS/BRZ.* In the main text, there is also a typographical error where the SUMO site on *BZR1* should read “K280” and “K310” instead of “K320.” The authors apologize for the errors.

**Figure 4:**
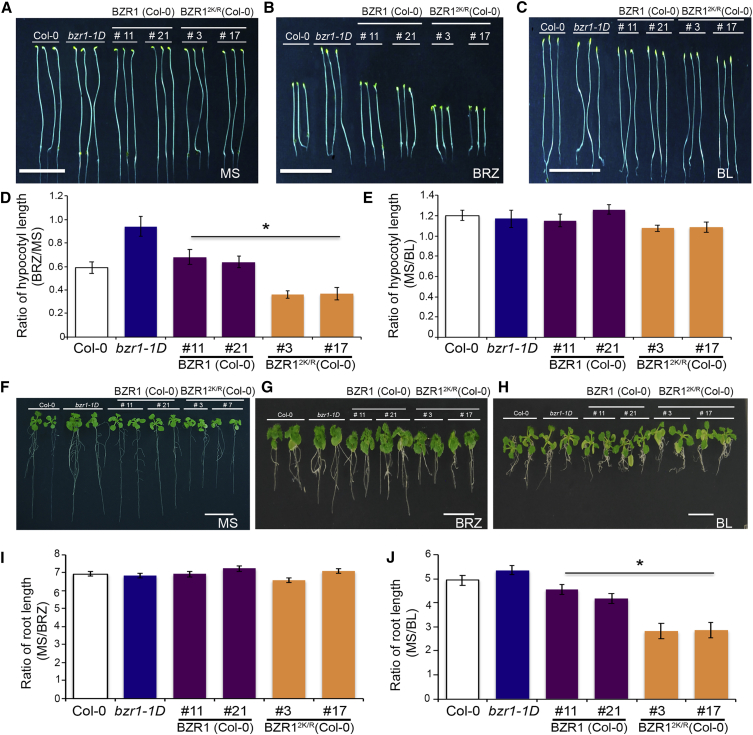
SUMOylated BZR1 Promotes BR-Mediated Plant Growth (Corrected)

**Figure 4:**
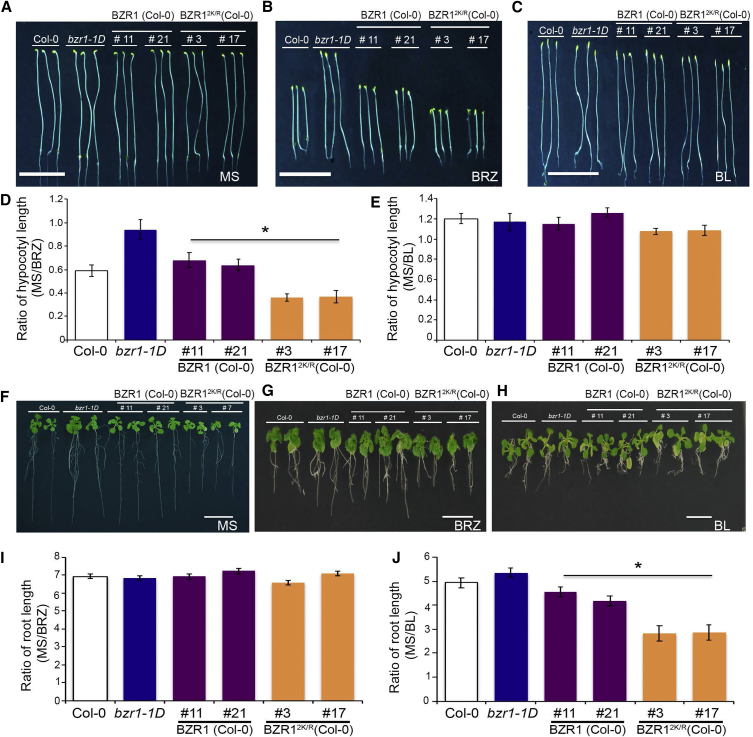
SUMOylated BZR1 Promotes BR-Mediated Plant Growth (Original)

