# Supplementary material for: SUMO Conjugation to BZR1 Enables Brassinosteroid Signaling to Integrate Environmental Cues to Shape Plant Growth
Source: Curr Biol. 2020 Apr 20;30(8):1410–1423.e3. doi: 10.1016/j.cub.2020.01.089 (PMC7181186; doi:10.1016/j.cub.2020.01.089)
Supplement: Document S1. Figures S1–S6 and Tables S1 and S2 [file mmc1.pdf]

Current Biology, Volume *30*

Supplemental Information

SUMO Conjugation to BZR1 Enables Brassinosteroid Signaling to Integrate Environmental Cues to Shape Plant Growth

Moumita Srivastava, Anjil K. Srivastava, Beatriz Orosa-Puente, Alberto Campanaro, Cunjin Zhang, and Ari Sadanandom

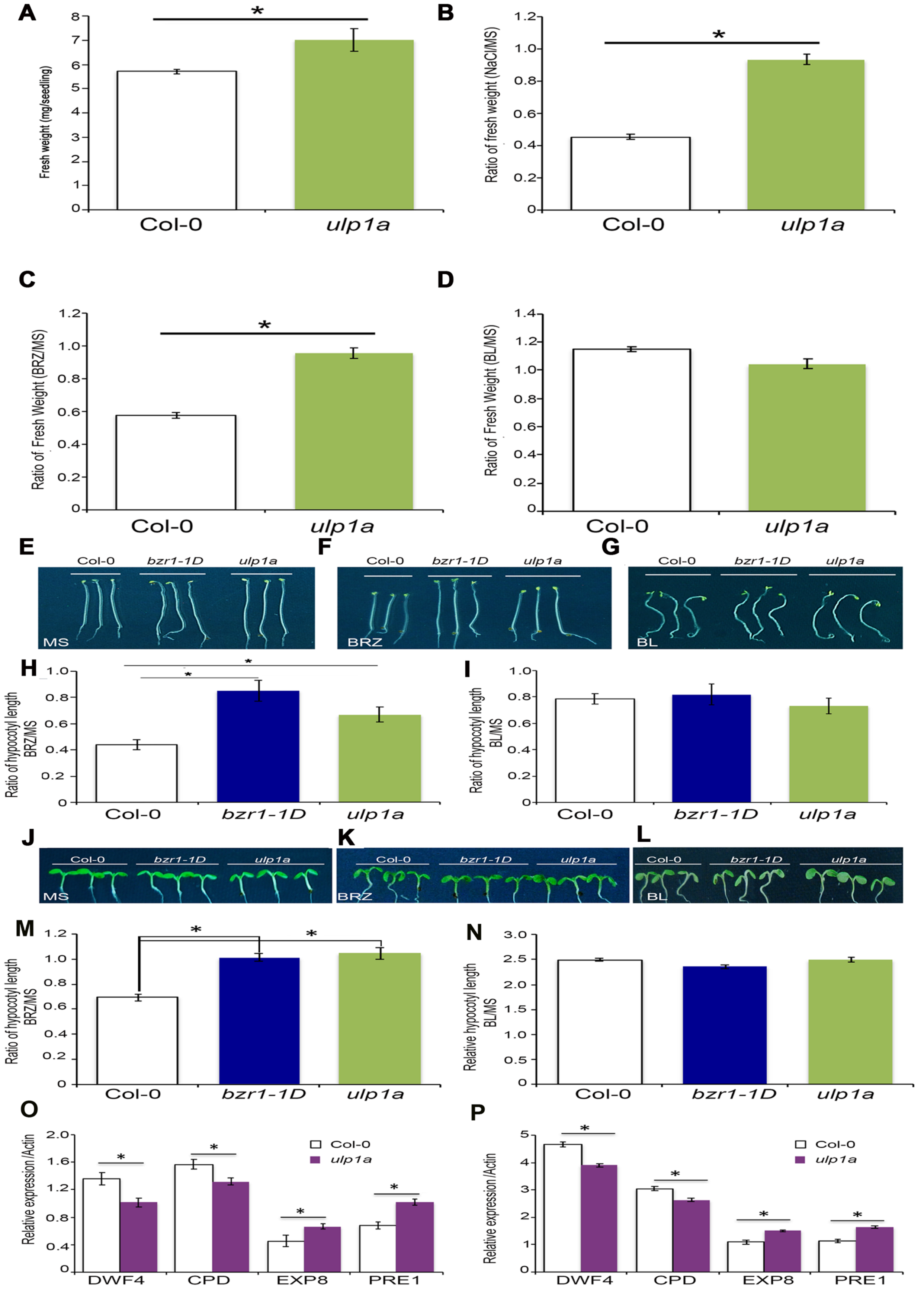

Figure S1; ULP1a is the SUMO protease involved in BR signaling. Related to Figure 1. (A) Quantification for fresh weight for Col-0 and *ulp1a* grown on MS for 12 days. (B-D) Quantification for relative fresh weight for 12d old Col-0 and *ulp1a* grown on BRZ (2μM) (B), BL(1μM) (C) and NaCl (100mM) with reference to untreated samples. *ulp1a* is less sensitive to BRZ mediated inhibition of seedling growth. (E-G) Representative image of 6d old seedlings of Col-0, *bzr1-1D* and *ulp1a* grown on ½ MS, BRZ (2μM) and BL(1μM) medium respectively in dark. (H-I). Quantification of relative root growth for 6d old seedlings treated with BRZ (2μM) (H) and BL(1μM) (I) in reference to untreated seedlings. (J-L). Representative image of 6d old seedlings of Col-0, *bzr1-1D* and *ulp1a* grown on ½ MS, BRZ (2μM) and BL(1μM) medium respectively in light. (M-N). Quantification of relative root growth for 6d old seedlings treated with BRZ (2μM) (M) and BL(1μM) (N) in reference to untreated seedlings. (O-P) qRT-PCR analysis for Brassinosteroid target genes in 10d old Col-0 and ulp1a seedlings in untreated (O) and BRZ treated (P) conditions.

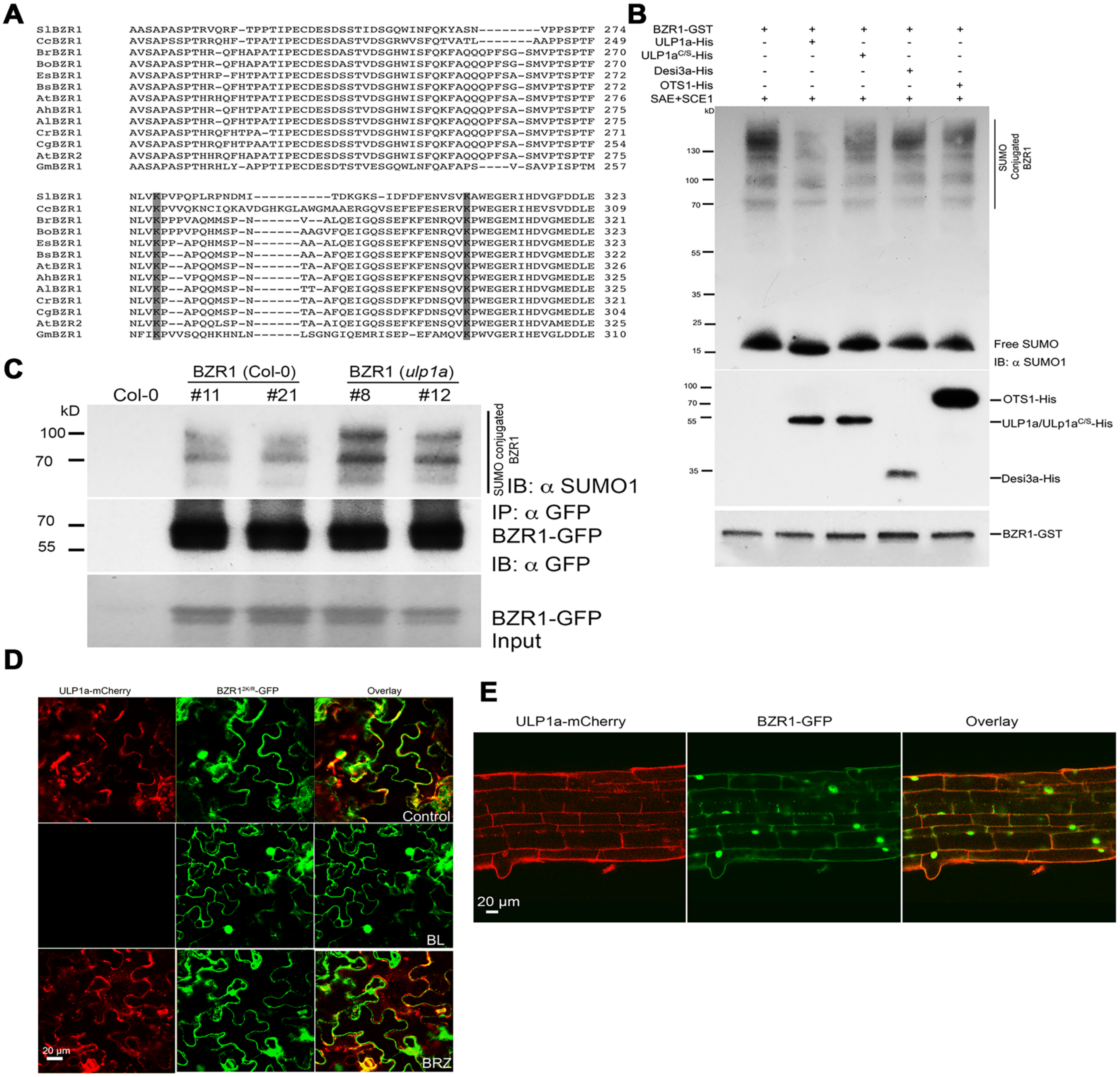

Figure S2, SUMO sites for BZR1 are conserved in different plant species and is deSUMOylated by the SUMO protease ULP1a. Related to Figure 2; (A) Amino acid alignment of BZR1 from Arabidopsis thaliana with *Solanum lycopersicum* (*Sl*BZR1), *Cajanus cajan* (CcBZR1), *Brassica rapa* (*Br*BZR1), Brassica olaracea (*Bo*BZR1), *Eutrema salsugineum* (*Es*BZR1), Boechera stricta (BsBZR1), Arabidopsis halleri (*Ah*BZR1), *Arabidopsis lyrata* (*Al*BZR1), *Capsella rubella* (*Cr*BZR1), Capsella grandiflora (*Cg*BZR1), *Arabidopsis thaliana* (*At*BZR2/BES1) and *Glycine max* (*Gm*BZR1). The conserved lysine at the two positions are highlighted in grey. Phytozome (http://phytozome.jgi.doe.gov/pz/portal.html) was used to obtain the BZR1 homolog sequences for different plant species. (B) ULP1a specifically deSUMOylates BZR1. High molecular weight conjugates of His-SUMO1 modified GST tagged BZR1 were formed by incubating purified SUMO E1 (SAE1 and 2) and E2 (SCE1) with BZR1-GST in the presence His-ULP1a, His-ULP1a$^{C/S}$, His-OTS1 and His-Desi3a and subsequently immunoblotted with anti-SUMO1 (αSUMO1) (upper panel) to detect SUMO chains, anti-His (αHis) (middle panel) to detect His-ULP1a, His-ULP1a$^{C/S}$, His-OTS1 and His-Desi3a and anti-GST (α GST) to detect BZR1-GST (lower panel). (C) BZR1 gets hyperSUMOylated in *ulp1a* background. Immunoprecipitation (IP: αGFP) experiments were carried out with anti-GFP beads from total protein derived from transgenic lines expressing $_{pro}$BZR1::BZR1-GFP expressed in Col-0 or $_{pro}$BZR1::BZR1-GFP expressed in ulp1a background. Immunoblots were probed with anti-GFP (IB: αGFP) or anti- SUMO1/2 (IB: αSUMO1) antibodies. (D) ULP1a colocalises with BZR1$^{2K/R}$ in the cytoplasm. *N. benthamiana* leaves co-infiltrated with ULP1a-mCherry and BZR1$^{2K/R}$-GFP were analyzed for fluorescence after 3 days. Before imaging, the leaves were infiltrated with 1μM BL or 2μM BRZ and incubated for 1 hour to see the effect of the treatments. Images were obtained using confocal laser scanning microscope Carl Zeiss Airyscan 880. Scale bar= 20μm. (E) BZR1 and ULP1a co-localise in stable transgenic lines in Arabidopsis. Confocal images of transgenic Arabidopsis seedlings co-expressing $_{pro}$BZR1::BZR1-GFP and 35S:ULP1a:mcherry.

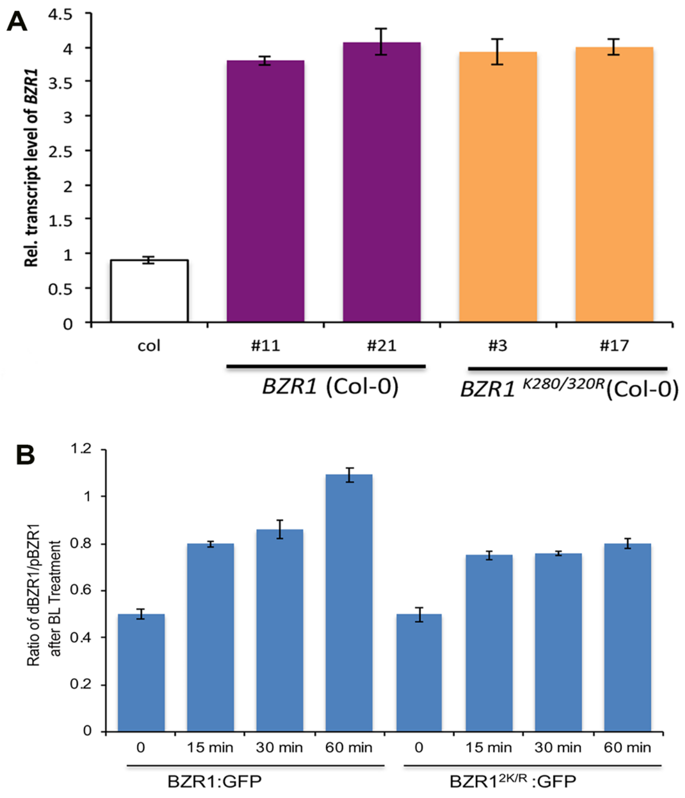

Figure S3, $_{pro}$BZR1::BZR1-GFP and $_{pro}$BZR1::BZR1$^{2K/R}$-GFP have similar transcript levels but respond differently in response to the brassinosteroids. Related to Figure 3. (A) Expression analysis of BZR1 in different transgenic lines. The expression levels of BZR1 in Col-0 and different transgenic plants by real-time PCR analysis. The experiment was replicated three times and data shown mean ± SD (N=3). (B) BL treatment does not affect the ratio of phosphorylated and dephosphorylated forms of BZR1 in $_{pro}$BZR1::BZR1$^{2K/R}$-GFP. Ratio of the band intensities for dephosphorylated BZR1 to phosphorylated BZR1 for BZR1 (Col-0) and BZR1$^{2K/R}$ (Col-0) when treated with BL at different time points (Figure 3E) quantified using image J software.

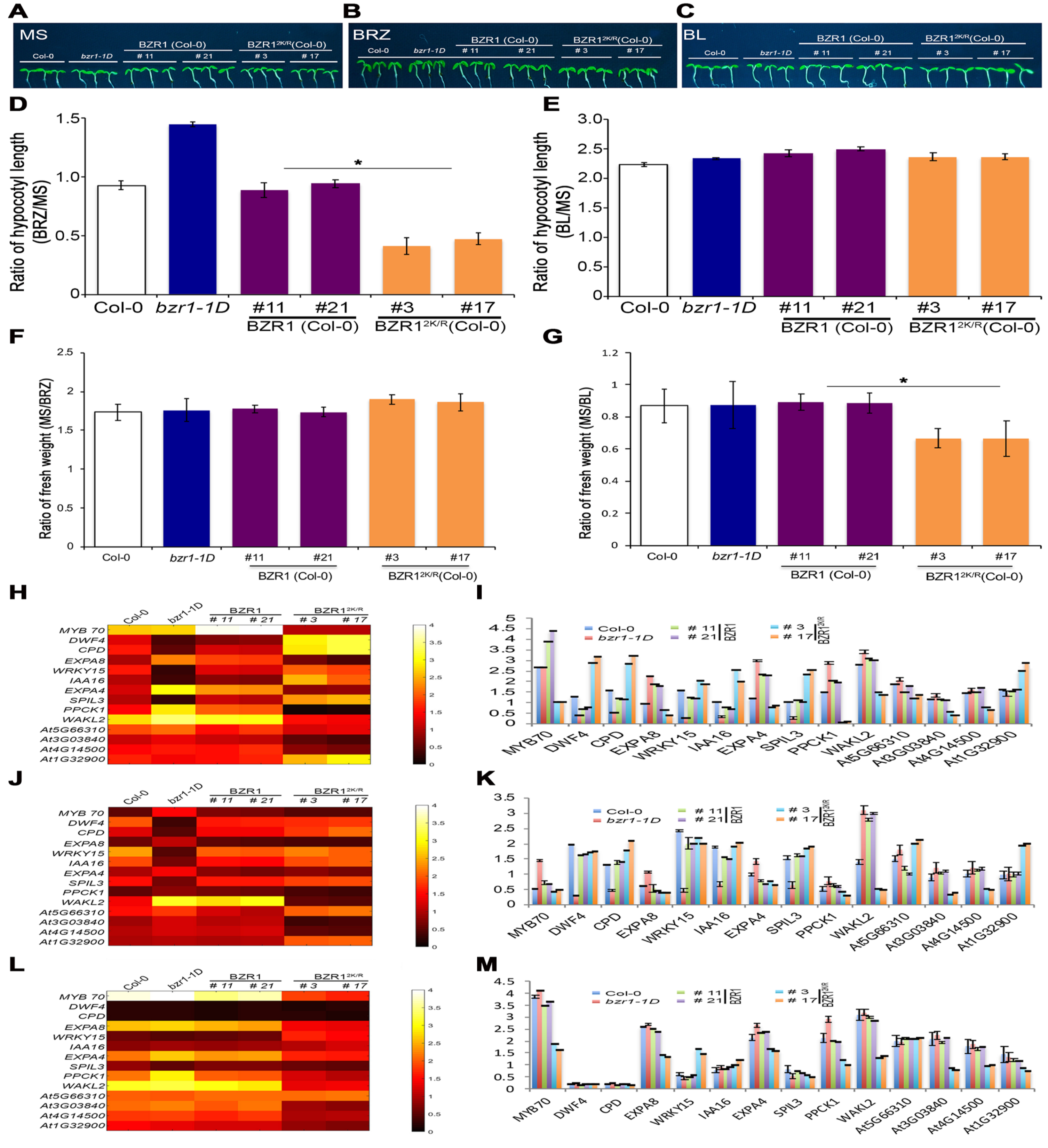

Figure S4; SUMO deficient form of BZR1 is hypersensitive to BR deficiency. Related to Figure 4. A-C. A-C Representative images of hypocotyl lengths of 6d old seedlings of Col-0, *bzr1-1D*, $_{pro}$BZR1::BZR1-GFP, $_{pro}$BZR1::BZR1$^{2K/R}$-GFP grown on ½ MS, BRZ (2µM) and BL (1µM) medium respectively in light. D-E. Relative growth of hypocotyls for seedlings grown in BRZ (D) or BL (E) with reference to the untreated seedlings. F-G Relative fresh weight for 10d old seedlings of Col-0, *bzr1-1D*, $_{pro}$BZR1::BZR1-GFP and $_{pro}$BZR1::BZR1$^{2K/R}$-GFP grown on BRZ (2µM) (F) and BL (1µM) (G) medium with reference to untreated seedlings. H-M. SUMO mutant form of BZR1 fails to function as a transcription factor. Heat map and quantification of the BL target genes for which the expression was studied in Col-0, bzr1-1D, transgenic $_{pro}$BZR1::BZR1-GFP and transgenic $_{pro}$BZR1::BZR1$^{2K/R}$-GFP transgenic lines without any treatment (H and I), on treatment with 2µM BRZ (J and K) and treated with 1µM BL (L and M). RNA was isolated from10 day old seedlings treated with respective treatments for 5 hours and used for cDNA preparation. Real Time PCR analysis was done which confirmed a defective transcription factor activity for the SUMO deficient form of BZR1.

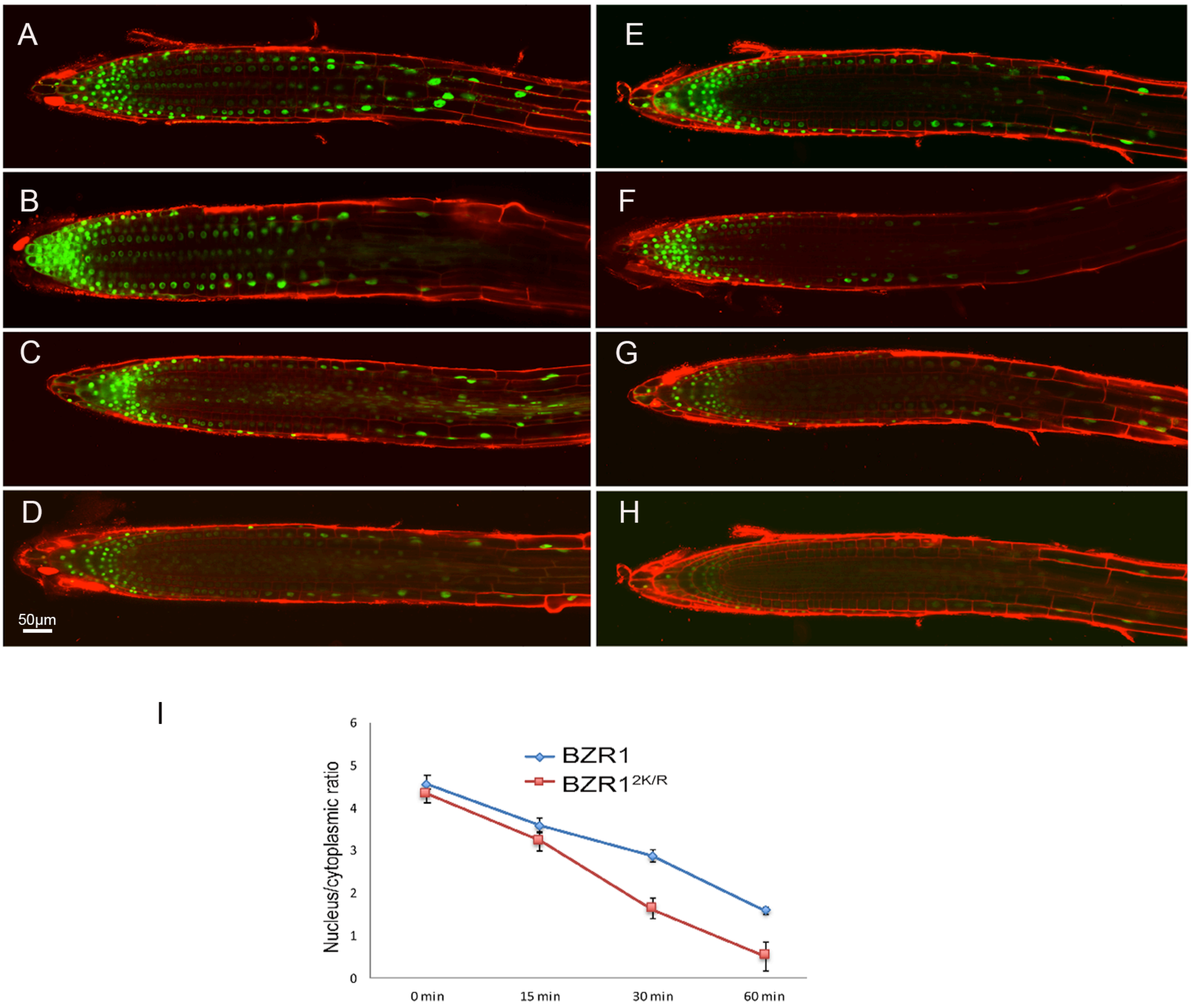

Figure S5; BRZ causes rapid degradation of $_{pro}$BZR1:BZR12K/R-GFP. Related to Figure 5. A-D. Confocal images of 4d old proBZR1::BZR1-GFP seedlings at 0, 15, 30 and 60 minutes of treatment with 2µM BRZ respectively. E-H. Confocal images of 4d old $_{pro}$BZR1::BZR1$^{2K/R}$-GFP seedlings at 0, 15, 30 and 60 minutes of treatment with 2µM BRZ respectively. I N/C ratios of the GFP signals from the elongation zone of the roots at 0, 15, 30 and 60 min time points after treatment with 2µM BRZ.

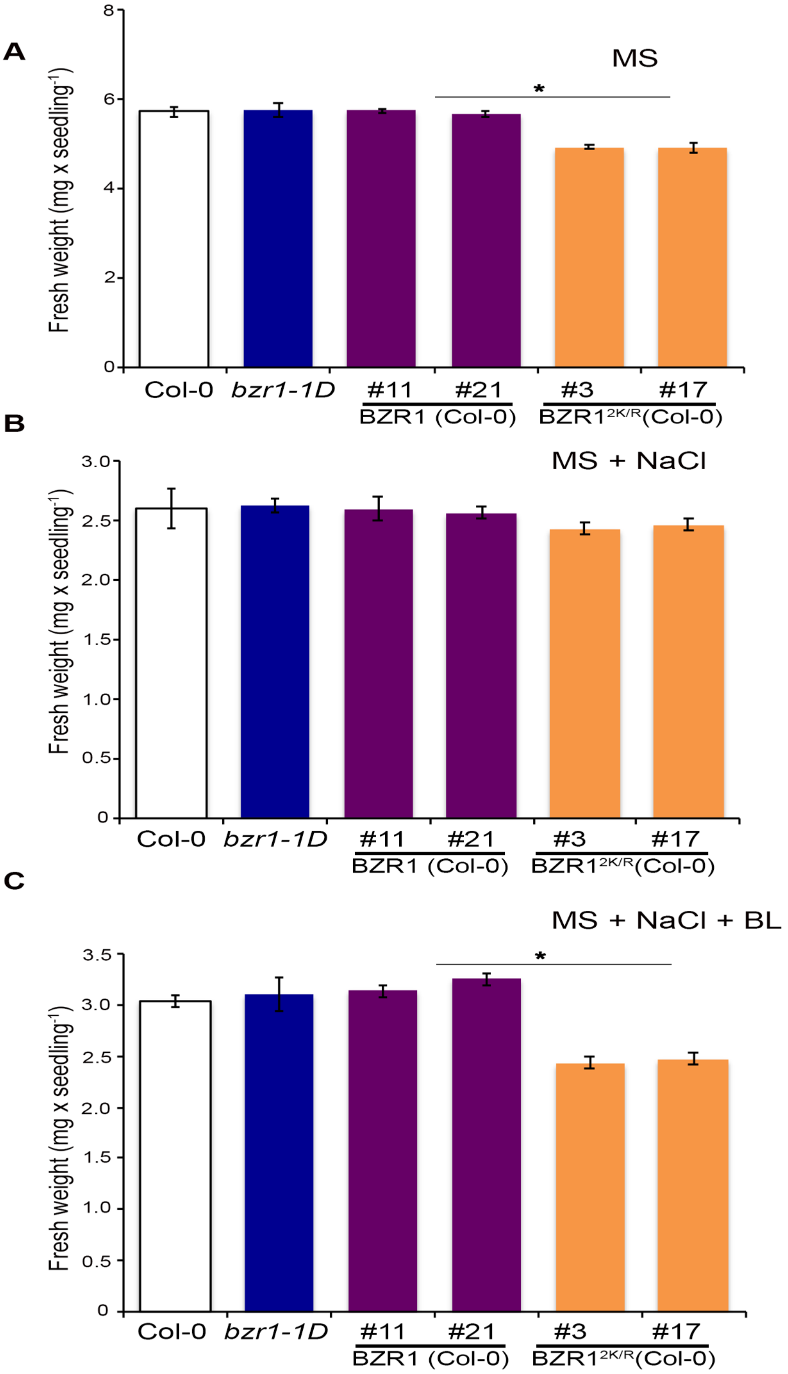

Figure S6; Brassinosteroids cannot rescue the short root phenotype for proBZR1::BZR1$^{2K/R}$-GFP seedlings. Related to Figure 7. (A) Quantification of fresh weight measurements for 12d old Col-0, *bzr1-1D*, $_{pro}$BZR1::BZR1-GFP and $_{pro}$BZR1::BZR1$^{2K/R}$-GFP plants grown on MS. (B) Quantification of fresh weight measurements of 12d old Col-0, *bzr1-1D*, $_{pro}$BZR1::BZR1-GFP and $_{pro}$BZR1::BZR1$^{2K/R}$-GFP plants grown on 100mM NaCl. (C) Quantification of fresh weight measurements of 12d old Col-0, *bzr1-1D,* $_{pro}$BZR1::BZR1-GFP and $_{pro}$BZR1::BZR1$^{2K/R}$-GFP plants grown on 100mM NaCl for 6 days and then on 1µM BL for next 6 days.

Genes	Forward primer	Reverse primer	Function
proBZR1	5'CACCGGTCTCAAGTAGCCTA ATTCATCGAACCCTCT 3'	5'TTTGGTCTCATCATCGGGAA AACCAACAACCAA 3'	Cloning
BZR1	5' CACCATGACTTCGGATGGAGC TACG 3'	5' ACCACGAGCCTTCCCATTTC 3'	Cloning
proBZR1::BZR1 overlap	5' CGTAGCTCCATCCGAAGTCAT CGGGAAAACCAACAACCAA 3'	5' TTGGTTGTTGGTTTTCCCGAT GACTTCGGATGGAGCTACG 3'	Cloning
BZR1K280R	5' TCAATCTTGTGAGACCTGCGC T 3'	5' AGGCGCAGGTCTCACAAGATT GA 3'	Site-directed mutagenesis
BZR1K320R	5' TCAAAGCTCTCAGTTTAAATTT GAGAATAG 3'	5' CTATTCTCAAATTTAAACTGAG AGCTTTG 3'	Site-directed mutagenesis
BZR1	5' GTTACCCCACCGGTCTCATC 3'	5' TGAAACTGGTGGCGATGTGT 3'	Genotyping
BIN2	CACCATGGCTGATGATAAGGA GATG	AGTTCCAGATTGATTCAAGAA GCTT	Cloning
ULP1a	5' CACCATGAAAAACCAATCTAG GG 3'	5' CTCGGCTTTCAGTTGCAGA 3'	Cloning
SAUR8	5' GGGACACTTCCCTGTCTACG 3'	5' GGAATGGTGAGACCCATGTCA 3'	Q-PCR
ulp1a	5′ GCCTTTTCAGAAATGGATAAA TAGCCTTGCTTCC 3′	5' CTCGGCTTTCAGTTGCAGA 3'	genotyping
SCE1	5' CACCATGGCTAGTG GAATC 3'	5' TTA GACAAGAGCA GGATAC 3'	Cloning
Actin	5' CCATCGCTCATCGGAATGGA 3'	5' TGGAACCACCACTGAGAACG 3'	Q-PCR
ULP1a	5' TGGTTACCACAAACGGAGCG 3'	5' TGAACTTGTCTCCGCAACGA 3'	Genotyping
MYB70	5' GGCGGAGGAAGATGATACGA 3'	5' ACAACTCTGCCCTTCCTCTC 3'	Q-PCR
DWF4	5' GGTGGAAAGTGTTACCGGTG 3'	5' TCCTCCAAACGGCATGTAGT 3'	Q-PCR
CPD	5' TCTCCCTCTCTTCTCCACCA 3'	5' GCGACAAGTAAAGCCACCAA 3'	Q-PCR
EXPA8	5'GTTTTACGGCGGCGAAGATG 3'	5'CCACCTCGGGTCATCGTTAC 3'	Q-PCR
WRKY15	5'GGCGGAGGAAGATGATACG A 3'	5' ACAACTCTGCCCTTCCTCTC3'	Q-PCR
IAA16	5'	5'	Q-PCR

	TGCTTGTAGGAGACGTACCG3’	CAACCGATCGAAACAGGCTT3’	
EXPA4	5’ AACTCCCTGAATCAGCCACA3’	5’ CCTTCGCCTTATCCGCAAAA3’	Q-PCR
SPIL3	5’ TGCCTCCTCCATGCATGTTA3’	5’ TGGGCTTATACGGGCTTTGA3’	Q-PCR
PPCK1	5’ AGGAGTGTCGGGCATGTAAA3’	5’ CATCGCCAAACTCAGAAGCA3’	Q-PCR
WAKL2	5’ AAGCTCCCGTAACTTCCTCC3’	5’ ATACCCGCCATAACAGCCTT3’	Q-PCR
At5G66310	5’ GGCCATCTGATGCTTCTGTG3’	5’ TTGCTGCGTCCTTGAAACTC3’	Q-PCR
At3G03840	5’ TTGCAGTGTACGTAGGGGAG3 ’	5’ TCTTCAGGACAAGGGATCGT3’	Q-PCR
At4G14500	5’ GGCCTTCACTCTGGGATCAT3’	5’ AACCACATGCCCTTCTCAGA3’	Q-PCR
At1G32900	5’ GCTGGTAAGATTGTGTGTGAG	5’ CACAACACAAGTGTCCCAAG	Q-PCR

Table S1: List of primers used in the study, Related to STAR methods

Genes	Constructs	Vectors	Experiment
BZR1	BZR1:GFP	pEG103	Co-IP/Confocal
$BZR1^{2K/R}$	$BZR1^{2K/R}$:GFP	pEG103	Co-IP/Confocal
proBZR1::BZR1	BZR1:GFP	pMDC107	Generation of transgenics/Confocal
proBZR1::$BZR1^{2K/R}$	$BZR1^{2K/R}$:GFP	pMDC107	Generation of transgenics/Confocal
BZR1	GST:BZR1	pDEST15	In-vitro pull down assay
BIN2	HA:BIN2	pEG201	Co-IP
BIN2	His:BIN2	pDEST17	In-vitro pull down assay
ULP1a	HA:ULP1a	pEG201	Co-IP
ULP1a	ULP1a:mcherry	pMDC43	Confocal
ULP1a	His:ULP1a	pDEST17	In-vitro pull down assay
ULP1a	HA:ULP1a	pGWB15	Generation of transgenics
SCE1(E2)	HA:SCE1	pEG201	Co-IP
SUMO1	His:SUMO1	pDEST17	Isopeptide cleaved SUMO chains

Table S2: List of constructs used in the study, Related to STAR Methods
