# Supplementary material for: SUMO Conjugation to BZR1 Enables Brassinosteroid Signaling to Integrate Environmental Cues to Shape Plant Growth
Source: Curr Biol. 2020 Apr 20;30(8):1410–1423.e3. doi: 10.1016/j.cub.2020.01.089 (PMC7181186; doi:10.1016/j.cub.2020.01.089)
Supplement: Document S2. Article plus Supplemental Information [file mmc2.pdf]

Article

Current Biology

SUMO Conjugation to BZR1 Enables Brassinosteroid Signaling to Integrate Environmental Cues to Shape Plant Growth

Graphical Abstract

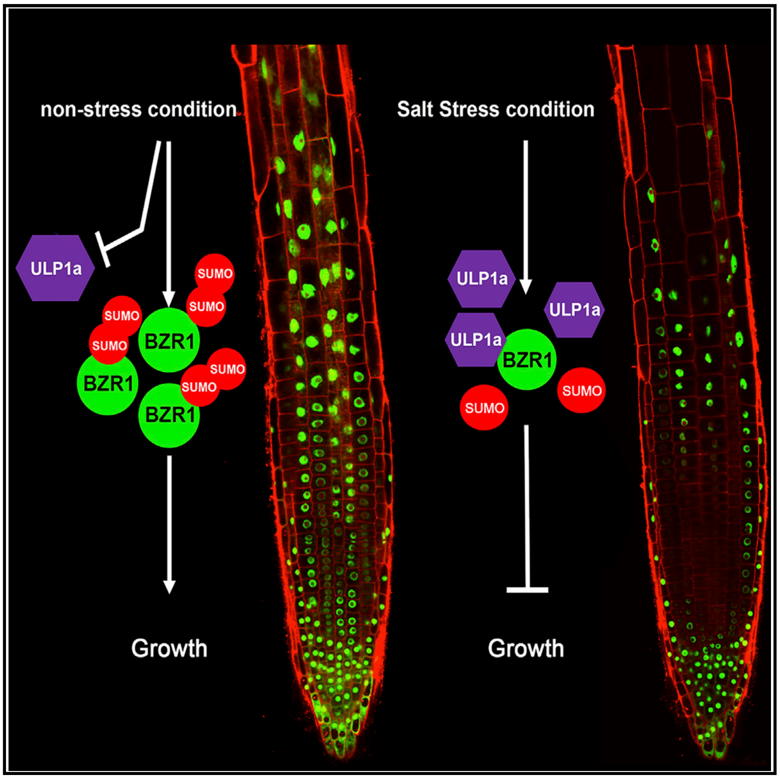

Authors

Moumita Srivastava,
Anjil K. Srivastava,
Beatriz Orosa-Puente,
Alberto Campanaro, Cunjin Zhang,
Ari Sadanandom

Correspondence

ari.sadanandom@durham.ac.uk

In Brief

SUMO conjugation is emerging as an important mechanism to transduce environmental cues into cellular signaling. Srivastava et al. reveal that SUMOylation stabilizes the BR-responsive transcription factor BZR1 to promote growth under non-stressed conditions. Salinity stimulates BZR1 deSUMOylation via ULP1a SUMO protease to suppress growth.

Highlights

- BZR1 SUMOylation allows brassinosteroids to shape plant growth to its environment
- SUMOylation stabilizes BZR1 by inhibiting BIN2 interaction, promoting plant growth
- Salinity stimulates BZR1 deSUMOylation via ULP1a SUMO protease to suppress growth
- BRs destabilize ULP1a, allowing SUMOylated BZR1 to accumulate and promote growth

Srivastava et al., 2020, Current Biology *30*, 1410–1423
April 20, 2020 © 2020 The Author(s). Published by Elsevier Ltd.
https://doi.org/10.1016/j.cub.2020.01.089

CellPress

Current Biology
Article

SUMO Conjugation to BZR1 Enables Brassinosteroid Signaling to Integrate Environmental Cues to Shape Plant Growth

Moumita Srivastava,[1] Anjil K. Srivastava,[1] Beatriz Orosa-Puente,[1] Alberto Campanaro,[1] Cunjin Zhang,[1] and Ari Sadanandom[1,2,*]
[1]Department of Biosciences, Durham University, Stockton Road, Durham DH1 3LE, UK
[2]Lead Contact
*Correspondence: ari.sadanandom@durham.ac.uk
https://doi.org/10.1016/j.cub.2020.01.089

SUMMARY

Brassinosteroids (BRs) play crucial roles in plant development, but little is known of mechanisms that integrate environmental cues into BR signaling. Conjugation to the small ubiquitin-like modifier (SUMO) is emerging as an important mechanism to transduce environmental cues into cellular signaling. In this study, we show that SUMOylation of BZR1, a key transcription factor of BR signaling, provides a conduit for environmental influence to modulate growth during stress. SUMOylation stabilizes BZR1 in the nucleus by inhibiting its interaction with BIN2 kinase. During salt stress, *Arabidopsis* plants arrest growth through deSUMOylation of BZR1 in the cytoplasm by promoting the accumulation of the BZR1 targeting SUMO protease, ULP1a. ULP1a mutants are salt tolerant and insensitive to the BR inhibitor, brassinazole. BR treatment stimulates ULP1a degradation, allowing SUMOylated BZR1 to accumulate and promote growth. This study uncovers a mechanism for integrating environmental cues into BR signaling to shape growth.

INTRODUCTION

Plants are constantly challenged with various environmental stresses during their life cycle. Therefore, plants have evolved effective stress-responsive mechanisms to tailor growth and development to match their immediate environment. Phytohormone-mediated signaling mechanisms coordinate a myriad of physiological processes to regulate growth and development in response to exogenous signals [1, 2]. Brassinosteroids (BRs) are a major class of plant-specific steroid hormones that modulate a variety of physiological processes from seed development to flowering and senescence [3–5]. BRs are perceived by plants by direct binding of the hormone to a membrane receptor kinase, BRI1 (brassinosteroid insensitive 1), that initiates a signal transduction cascade involving a series of phosphorylation and dephosphorylation steps. Initially, the binding of BRs causes the recruitment of the co-receptor kinase, BAK1 (BRI1-associated kinase 1), to BRI1 [6–11]. Upon receptor BAK1-BRI1 complex formation, BRI1 phosphorylates BSK1 (BRI1 substrate kinase 1) and CDG1 (constitutive differential growth 1), which in turn, bind to and phosphorylate BSU1 (BRI1 suppressor 1) kinase [12–14]. BSU1 then inactivates BIN2, a GSK3-like kinase, by dephosphorylating a conserved tyrosine residue within this protein [15]. In absence or low levels of BRs, BIN2 is predominantly dephosphorylated and activated to phosphorylate two homologous transcription factors, BZR1 and BES1, and cause their cytoplasmic retention, where they are targeted for degradation by proteasome [16–19]. In the presence of BRs, BIN2 is inactivated by BSU1 and degraded by the proteasome [20]. BZR1 and BES1 are then dephosphorylated by a protein phosphatase PP2A [21] and move into the nucleus, where they bind to promoters of target genes, leading to the expression of the BR-regulated genes that enact physiological responses in plants [22–25]. Evidence to date indicates that, after perception of BRs, the inactivation of BIN2 that targets BZR1/BES1 transcription factors remains the major point of regulation in BR signaling. However, little is known of the critical steps that facilitate BIN2 interaction with these transcription factors that ultimately determines BR signaling in plants.

Posttranslational modification of the proteome has been established as a critical step in the rapid reprogramming of cellular signaling during biotic and abiotic stresses. Modification of proteins by small ubiquitin-like modifier (SUMO) is emerging as an important mechanism to swiftly refocus molecular pathways during plants' stress signaling [26, 27]. SUMO is synthesized as an inactive precursor and requires SUMO proteases to cleave the C-terminal tail to form mature SUMO. This occurs by exposure of a di-glycine motif, where target attachment occurs in a series of enzymatic reactions very similar to ubiquitination, which includes activation, conjugation, and ligation [28–30]. Upon covalent conjugation to target proteins, SUMO affects protein-protein interactions, subcellular localization, and stability of target proteins [31, 32]. In the BR pathway, SUMOylation of the basic-helix-loop-helix (bHLH) transcription factor CESTA regulates plant development by coordinating CESTA stability and nuclear localization [33]. Nevertheless, SUMO conjugation to target proteins in plants is largely observed only upon environmental stress; therefore, we postulate that SUMO may provide the environmental input to modulate BR responses, but how this may be achieved is not known. SUMOylation is reversible, as SUMO-specific proteases can cleave SUMO from their target proteins, thereby

 © 2020 The Author(s). Published by Elsevier Ltd.
This is an open access article under the CC BY license (http://creativecommons.org/licenses/by/4.0/).

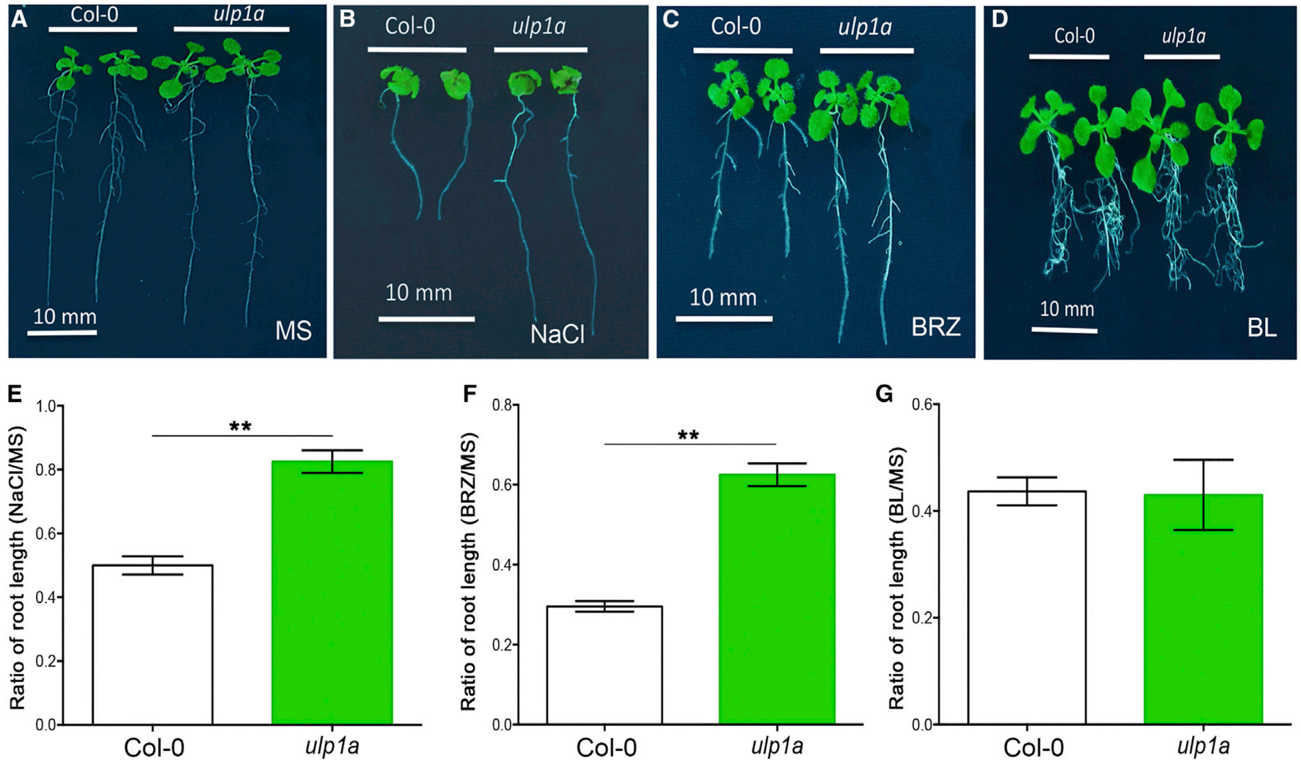

Figure 1. ULP1a Is the SUMO Protease Required to Suppress Growth during Salt Stress in *Arabidopsis*

(A) Representative image of root lengths of 12-day-old young adult plants of Col-0 and ul*p1a* grown on ½ Murashige and Skoog.

(B and E) Representative image of root lengths of 12-day-old young adult plants of Col-0 and ul*p1a* grown on 100 mM NaCl (B) and quantification of relative root growth in presence of salt with respect to untreated plants (E).

(C and F) Representative image of root lengths of 12-day-old young adult plants of Col-0 and ul*p1a* grown on BRZ (2 μM) medium (C) and quantification of root lengths in presence of the treatment with reference to untreated samples (F).

(D and G) Representative image of root lengths of 12-day-old young adult plants of Col-0 and ul*p1a* grown on BL (1 μM) medium (D) and quantification of root lengths in presence of the treatment with reference to untreated samples (G).

Scale bar, 1 cm.

Error bars indicate SE (n = 20). Asterisks indicate significant differences from Col-0. See also Figure S1.

potentially providing adaptability to signaling pathways that respond to changing environments [32].

In this study, we show that SUMOylation of BZR1 provides a conduit for environmental influence on BR signaling to modulate growth under salt stress. SUMOylation of BZR1 inhibits its interaction with BIN2. We identify the sites of SUMO modification in BZR1 and show that it is required for BZR1 protein to accumulate in the nucleus during BR signaling. *Arabidopsis* plants containing non-SUMOylatable BZR1 show impaired BR response post-salt stress, indicating that SUMOylation represents a critical step for environmental input into BR signaling. The SUMO protease ULP1a targets BZR1 for deSUMOylation in the cytoplasm. ULP1a mutants are more salt tolerant and insensitive to the BR inhibitor, BRZ. Exogenous BR treatment stimulates ULP1a degradation, therefore allowing SUMOylated BZR1 to accumulate to promote BR responses. We demonstrate that, during salt stress, ULP1a accumulates to generate deSUMOylated BZR1, which is more unstable, therefore attenuating BR-promoted growth in *Arabidopsis*. This study uncovers a new facet of BR signaling that integrates environmental conditions with plant growth and development.

RESULTS

ULP1a SUMO Protease Suppresses Growth during Salt Stress in *Arabidopsis*

In eukaryotes, posttranslational modifications, such as SUMOylation, underpin major mechanisms employed to tune physiological responses to suit environmental conditions. SUMO proteases are pivotal components of the SUMOylation cycle in plants [26, 27, 34–36]. Previously, *Arabidopsis* mutant seedlings lacking SUMO proteases OTS1 and OTS2 were shown to exhibit reduced root growth in response to salt stress [27], indicating a role for these SUMO proteases in promoting growth under stress. We speculated that analogous SUMO proteases might operate to repress growth during stress as part of a fine-tuning mechanism. To this end, we identified the *Arabidopsis* SUMO protease mutant, ul*p1a* [36], which showed increased seedling root growth when compared to Col-0 (wild-type [WT]) under salt stress (Figures 1A, 1B, 1E, S1A, and S1B). Additionally, ul*p1a* mutants had larger shoots when fresh weights were compared to WT, even on Murashige and Skoog-only plates (Figure S1A).

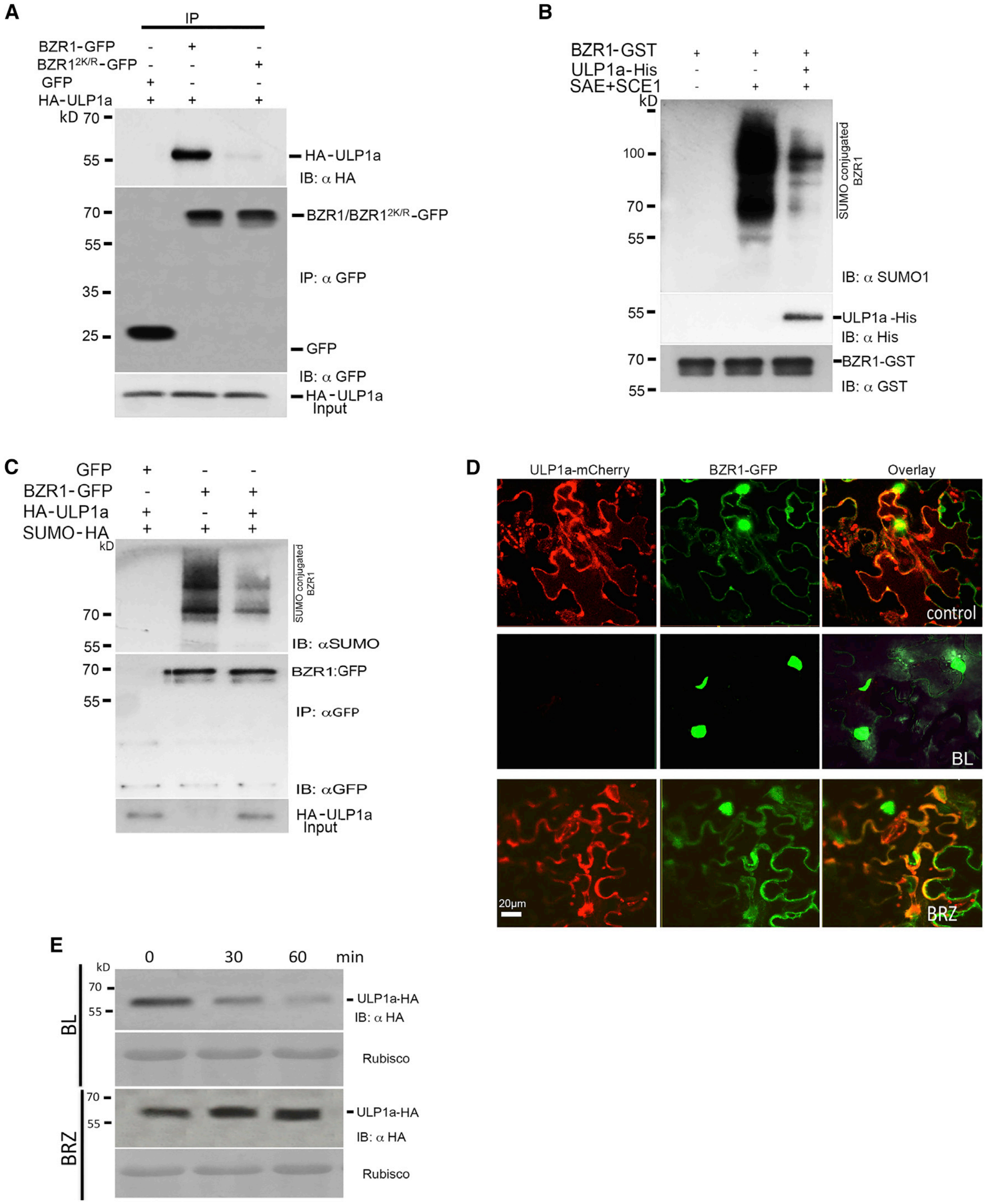

Figure 2. ULP1a Targets BZR1 for DeSUMOylation

(A) BZR1-GFP interacts with HA-ULP1a. *N. benthamiana* leaves transiently expressing BZR1-GFP or BZR1$^{2K/R}$-GFP with HA-ULP1a were collected for immunoprecipitation. Subsequently, total protein was subjected to immunoprecipitation with anti-GFP immunoaffinity beads (IP: αGFP) followed by immunoblot analysis with anti-HA (IB: αHA) antibodies to detect HA-ULP1a and αGFP (IB: αGFP) antibodies to detect BZR1-GFP. Total protein of all samples was probed with anti-HA antibody to determine ULP1a protein levels (HA-ULP1a input). GFP was used as a negative control.

(B) ULP1a is a bona fide SUMO protease that deSUMOylates BZR1 *in vitro*. High-molecular-weight conjugates of His-SUMO1-modified, GST-tagged BZR1 were formed by incubating purified SUMO E1 (SAE1 and 2) and E2 (SCE1) with BZR1-GST in the presence or absence of His-ULP1a and subsequently immunoblotted with anti-SUMO1 (αSUMO1) (upper panel) to detect SUMO chains, anti-His (αHis) (middle panel) to detect His-ULP1a, and anti-GST (αGST) to detect BZR1-GST (lower panel).

(C) *N. benthamiana* leaves transiently expressing BZR1-GFP and SUMO-HA with or without HA-ULP1a were collected for immunoprecipitation. Subsequently, total protein was subjected to immunoprecipitation with αGFP immunoaffinity beads (IP: αGFP) followed by immunoblot analysis with αSUMO1 (IB: αSUMO1)

(legend continued on next page)

It is known that salt stress inhibits BR signaling to repress growth [37–39]; we wanted to ascertain whether ul*p1a* mutants were sensitive to brassinazole (BRZ) that inhibits the biosynthesis of brassinosteroids [40]. Although Col-0 seedling showed reduced growth in BRZ, ul*p1a* was observed to be less sensitive to BRZ (Figures 1C, 1F, S1C, S1F, S1H, S1K, and S1M). However, no phenotypic difference was observed in the presence of growth-promoting BL (epi-brassinolide) in any of the genotypes (Figures 1D, 1G, S1D, S1G, S1I, S1L, and S1N). These data indicate that ULP1a has a clear role in inhibiting growth under salt stress and ULP1a influences BR signaling. We next studied the effect of the ULP1a mutation on the expression of the brassinosteroid-regulated genes. Brassinosteroid availability is known to suppress the expression of BR biosynthetic genes, such as *DWF4* and *CPD*, via BZR1 transcription factor, which provides a feedback-regulation loop to maintain homeostatic levels of BR [24], whereas the availability of BRs activates the expression of many other target genes, such as *EXP8*, *PRE1*, and *PER5* [23]. Quantitative RT-PCR analysis shows that ul*p1a* mutants have reduced levels of *DWF4* and *CPD* when compared to Col-0 (Figures S1O and S1P). Additionally, ul*p1a* mutants showed an elevated level of the expression of the target genes activated by BRs (Figures S1O and S1P). Thus, our data reveal that ULP1a plays a role in suppressing BR signaling.

ULP1a Regulates BR Signaling by Directly Targeting BZR1 for DeSUMOylation in the Cytoplasm

SUMO proteases regulate signaling pathways by promoting deconjugation of SUMO from their target substrates [41]. Once establishing that ULP1a impacts BR responses, we performed *in silico* analysis of the components of the BR signaling pathway to identify potential SUMO targets and found BZR1, a positive regulator of BR-mediated growth responses, to have two putative high-confidence SUMO conjugation sites at lysines 280 and 320, which were conserved across BZR1 homologs from different plant species (Figure S2A), indicating that ULP1a may target BZR1 for deSUMOylation as a means to regulate BR signaling. First, we wanted to ascertain whether there is any physical interaction between BZR1 and ULP1a. We performed co-immunoprecipitation experiments in *N. benthamiana* via Agrobacterium-mediated transient assays with epitope-tagged BZR1-GFP and hemagglutinin (HA)-ULP1a. Immunoblotting with anti-HA and anti-GFP antibodies showed that BZR1 co-immunoprecipitated with ULP1a (Figure 2A), indicating that BZR1-ULP1a physically interact although the non-SUMO double lysine to arginine mutant (BZR1$^{2K/R}$-GFP) only interacted weakly. We also performed *in vitro* deSUMOylation assays to confirm that ULP1a directly targets SUMOylated BZR1 for deconjugation. Addition of purified histidine-tagged ULP1a to SUMOylated glutathione S-transferase (GST)-BZR1, purified from bacterial SUMO expression system, reduced the level of SUMOylated GST-BZR1 (Figure 2B). In order to confirm the specificity of ULP1a activity against BZR1, the catalytic site mutant of ULPa (ULP1a$^{C/S}$), OTS1 and Desi3a SUMO proteases were also used in deSUMOylation assays (Figure S2B). We observed that only ULP1a could deSUMOylate BZR1, confirming that BZR1 was specifically deSUMOylated by ULP1a. To validate that ULP1a directly targets SUMO-conjugated BZR1 for deSUMOylation *in vivo*, we co-expressed SUMO-HA in the presence or absence of ULP1a in *N. benthamiana* transient assays. This transient gain-of-function analysis demonstrated that ULP1a targets SUMOylated BZR1 for deSUMOylation *in planta* (Figure 2C). As ULP1a was demonstrably the SUMO protease responsible for deSUMOylation of BZR1, we wanted to ascertain whether BZR1 should be hyper-SUMOylated in the absence of ULP1a. For this, we generated $_{pro}$*BZR1::BZR1:GFP* in the ul*p1a* loss-of-function mutant background and compared the SUMO levels of BZR1 in Col-0 and ul*p1a* backgrounds (Figure S2C). As expected, we observed that BZR1 is hyperSUMOylated in ul*p1a* background, further confirming that ULP1a is the SUMO protease that targets BZR1 for deSUMOylation. As ULP1a was localized in the cytoplasm and BZR1 was known to localize in both cytoplasm and nucleus [36, 42], we performed confocal microscopy in *N. benthamiana* using Agrobacterium-mediated transient assays to ascertain whether the two proteins colocalize in the cytoplasm. Fluorescence analysis showed that GFP-tagged BZR1 colocalized with m-Cherry-tagged ULP1a signals in the cytoplasm (Figure 2D, upper panel). Upon BL treatment, as expected, BZR1 accumulates in the nucleus; however, strikingly, fluorescence from ULP1a-mCherry was not observed, suggesting that ULP1a is not stable in the presence of BL (Figure 2D, middle panel). Nevertheless, the leaf tissues treated with BRZ showed a strong overlap of cytoplasmic fluorescence of the two proteins (Figure 2D, lower panel). Next, we wanted to ascertain the colocalization pattern for BZR1$^{2K/R}$-GFP with ULP1a-mCherry (Figure S2D). Fluorescence analysis showed that BZR1 colocalizes with ULP1a in the cytoplasm (Figure S6, upper panel). However, upon BL treatment, BZR1$^{2K/R}$-GFP was observed in the nuclei and in the cytoplasm although the WT BZR1 was only observed in the nuclei after BL treatment (Figure 2D), suggesting that SUMO plays a role in the appropriate localization of BZR1 (Figure S2D, middle panel). When treated with BRZ, BZR1$^{2K/R}$-GFP showed cytoplasmic localization, as expected (Figure S2D, lower panel). We also wanted to determine the colocalization of BZR1 and ULP1a in transgenic plants. For this, we generated stable transgenic *Arabidopsis* lines co-expressing $_{pro}$*BZR1::BZR1:GFP* and *35S::ULP1a-mCherry.* Confocal images of the transgenic seedlings show that both BZR1-GFP and ULP1a-mCherry

antibodies to detect HA-SUMO and αGFP (IB: αGFP) antibodies to detect BZR1-GFP. Equal amounts of HA-ULP1A protein were ascertained by probing with anti-HA antibodies. GFP was used as a negative control.

(D) ULP1a colocalizes with BZR1 in the cytoplasm. *N. benthamiana* leaves co-infiltrated with ULP1a-mCherry and BZR1-GFP were analyzed for fluorescence after 3 days. Before imaging, the leaves were infiltrated with 1 μM BL or 2 μM BRZ and incubated for 1 h to see the effect of the treatments. Images were obtained using confocal laser scanning microscope Carl Zeiss Airyscan 880. Scale bar, 20 μm.

(E) The accumulation of ULP1a protein is affected by brassinosteroid availability. 10-day-old *35S::ULP1a-HA* transgenic *Arabidopsis* seedlings were treated with a combination of 200 μM cycloheximide + 1 μM BL or 2 μM BRZ. Total proteins extracted at indicated time points were immunoblotted with anti-HA (IB: αHA) antibody.

See also Figure S2.

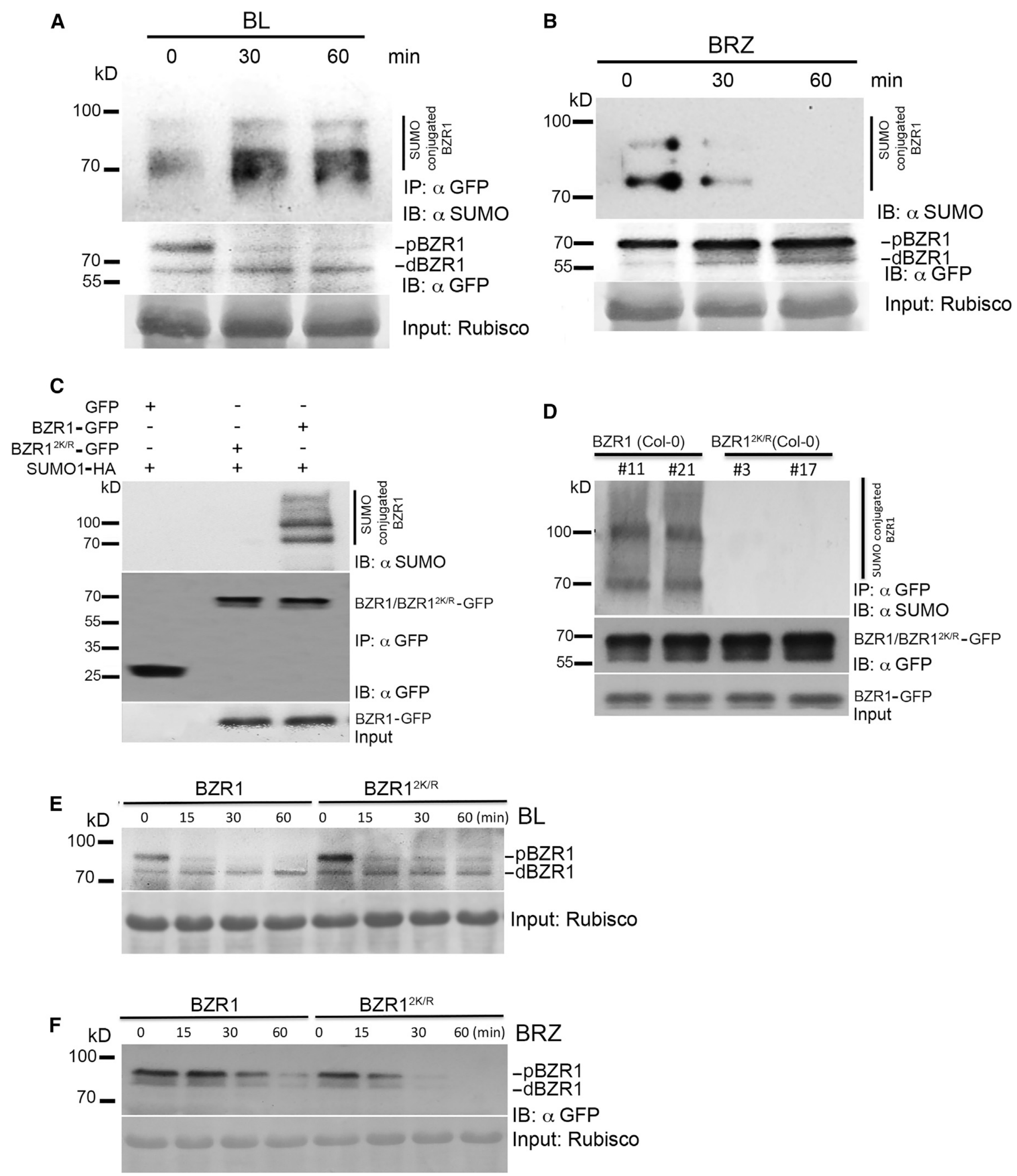

Figure 3. Brassinosteroids Promote BZR1 SUMOylation and ULP1a Degradation

(A) BL induces BZR1 SUMOylation. Immunoprecipitation (IP:αGFP) experiments were carried out with αGFP beads from total proteins derived from transgenic lines expressing $_{pro}$*BZR1::BZR1-GFP* in Col-0 background after treatment with 1 μM BL at time points of 0, 30, and 60 min. Immunoblots were probed with αGFP (IB: αGFP) or αSUMO1/2 (IB: αSUMO1) antibodies.

(B) BRZ inhibits SUMOylation of BZR1. Immunoprecipitation (IP: αGFP) experiments were carried out with αGFP beads from total proteins derived from transgenic lines expressing $_{pro}$*BZR1::BZR1-GFP* in Col-0 background after treatment with 2 μM BRZ at time points of 0, 30, and 60 min. Immunoblots were probed with αGFP (IB: αGFP) or αSUMO1/2 (IB: αSUMO1) antibodies.

(C) BZR1 is SUMOylated *in planta*. Transiently co-expressed BZR1-GFP or BZR1$^{2K/R}$-GFP transiently co-expressed with SUMO1-HA in leaves of *N. benthamiana* is shown. Immunoprecipitation (IP: αGFP) experiments were carried out with αGFP beads from total protein derived from these leaves. Immunoblots were probed with αGFP (IB: αGFP) or αSUMO1/2 (IB: αSUMO1) antibodies. GFP was used as a negative control.

(D) BZR1 is SUMOylated in stable *Arabidopsis* transgenic lines. Immunoprecipitation (IP: αGFP) experiments were carried out with αGFP beads from total protein derived from transgenic lines expressing $_{pro}$*BZR1::BZR1-GFP* or $_{pro}$*BZR1::BZR1$^{2K/R}$-GFP* in Col-0 background. Immunoblots were probed with αGFP (IB: αGFP) or αSUMO1/2 (IB: αSUMO1) antibodies. See also Figure S3.

(legend continued on next page)

colocalize in the cytoplasm (Figure S2E). Intrigued by this observation and to further understand the relationship between ULP1a SUMO protease, BZR1 SUMOylation, and BR signaling, we treated 10-day-old transgenic lines expressing 35S::HA-ULP1a with BL and BRZ at different time points. BL treatment promoted the degradation of ULP1a, whereas the protein accumulates upon BRZ treatment (in the presence of cycloheximide; Figure 2E), emphasizing a strong correlation between ULP1a protein stability and thus its deSUMOylation activity on BZR1 that directly impacts BR signaling. Our data reveal ULP1a as a new component of BR signaling. Taken together, these experiments demonstrate that ULP1a, the SUMO protease localized in the cytoplasm, deconjugates SUMO from BZR1 in the cytoplasm during BL-deficient conditions.

Brassinosteroids Promote BZR1 SUMOylation and Accumulation

To investigate whether BR signaling promoted SUMOylation of BZR1, we treated the 16-day-old dark grown $_{pro}$*BZR1::BZR1:GFP* seedling with brassinolide to determine the SUMO status of BZR1 using anti-SUMO1 antibodies [43]. We observed an increase in the pool of SUMOylated BZR1 protein with comparable amount of protein upon treatment with BL (Figure 3A), whereas BRZ treatment stimulated the deSUMOylation of BZR1 protein within 1 h of BRZ treatment (Figure 3B), which coincided with the accumulation of ULP1a (Figure 2E). Our data indicate that BR treatment promotes the rapid SUMOylation of BZR1 although inhibiting BR signaling triggers BZR1 deSUMOylation by increasing the abundance of ULP1a SUMO protease.

Transient assays in *N. benthamiana* leaves followed by immunoblot analysis with anti-SUMO1 antibodies [27] revealed that 35S promoter-driven, GFP-tagged BZR1 (35S::BZR1:GFP) is conjugated to *At*SUMO1, but the non-SUMO mutant (35S::BZR1$^{2K/R}$-GFP) is not (Figure 3C), validating the hypothesis that these conserved lysines are the sites for SUMO conjugation to BZR1 protein. Immunoblot analysis with anti-SUMO1 antibodies after immunoprecipitating own promoter-driven BZR1/BZR1$^{2K/R}$ from *Arabidopsis* transgenic plants confirmed this finding (Figure 3D). To rule out transcriptional variation between the different transgenic lines, the mRNA levels of the WT and SUMO variants of BZR fusion proteins were ascertained to be comparable (Figure S3A).

We also performed immunoblot analysis to study the effect of SUMOylation on the stability of BZR1 protein upon BL and BRZ treatment. As a result of the BL treatment, there was a gradual increase in the levels of dephosphorylated BZR1, whereas the phosphorylated BZR1 did not accumulate at different time points (Figures 3E and S3B). The SUMO-deficient form of BZR1 (BZR1$^{2K/R}$) did not show any difference between the change in the intensities of the dephosphorylated and the phosphorylated forms of BZR1 after 15 min of BL treatment (Figures 3E and S3B). On the other hand, treatment with BR inhibitor BRZ caused a rapid degradation of non-SUMO BZR1$^{2K/R}$-GFP protein levels compared to WT BZR1-GFP (Figure 3F). These data strongly indicate that SUMOylation plays an important role in BZR1 protein accumulation.

SUMOylated BZR1 Promotes Plant Growth

To determine the physiological effect of SUMOylation on BZR1, we used two independent transgenic lines that express BZR1 and BZR1$^{2K/R}$ with comparable level of transcripts under the control of the native promoter in Col-0 to first examine hypocotyl elongation in the dark and seedling root growth in the light under BL and BRZ treatment. All the dark-grown transgenic seedlings showed similar hypocotyl elongation compared to Col-0 seedlings in Murashige and Skoog media as well as in the presence of BL (Figures 4A and 4C). Similarly, no phenotypic differences were observed in light in either condition (Figures S4A and S4C). However, the hypocotyl elongation growth of *BZR1*$^{2K/R}$ transgenic lines was significantly inhibited in the presence of the BR inhibitor BRZ, unlike Col-0 and *BZR1* WT transgenic lines or the dominant positive *bzr1-1D* mutant (Figure 4B). We observed a similar inhibition of hypocotyl elongation in light grown seedlings of *BZR1*$^{2K/R}$ transgenic lines in BRZ (Figure S4B). We further investigated whether BR-mediated root growth is modulated by SUMO conjugation to BZR1. We analyzed root growth using the same transgenic lines in response to BRZ and BL in the medium and observed a clear association of SUMOylation of BZR1 and increased root growth. Plant carrying a non-SUMOylatable mutant of BZR1 showed reduced root growth compared to WT BZR1, Col-0, or the dominant positive mutant *bzr1-1D*, even in Murashige and Skoog-only media, indicating that SUMOylation of BZR1 is required to promote normal root growth (Figure 4F). In the presence of BRZ, *BZR1*$^{2K/R}$ transgenic seedling roots show similar growth inhibition compared to the other genotypes (Figure 4I). However, after BL treatment, *BZR1*$^{2K/R}$ seedlings show lesser root sensitivity (as measured by the ratio of root growth inhibition in Murashige and Skoog versus BL treatment; Figure 4J). Because higher concentrations of BR (used in the experiment) have an inhibitory effect on root growth [14], resistance of *BZR1*$^{2K/R}$ roots to BL indicate defective BR signaling in the SUMO-deficient BZR1 seedlings.

Surprisingly, $_{pro}$*BZR1::BZR1*$^{2K/R}$*-GFP* transgenic seedlings in the Col-0 background showed inhibited root growth phenotype, suggesting that BZR1$^{2K/R}$-GFP was acting in a dominant-negative manner. Therefore, we wanted to ascertain the activity of the endogenous BZR1 levels in these lines by analyzing the expression levels of the downstream target genes of the BZR1 transcription factor. We analyzed the expression levels of fourteen of the target genes for BL signaling from the RNAs isolated from Col-0, *bzr1-1D*, $_{pro}$*BZR1::BZR1-GFP*, and $_{pro}$*BZR1::BZR1*$^{2K/R}$*-GFP* transgenic lines after treatment with Murashige and Skoog, BL, or BRZ. To investigate the extent of BZR1 SUMOylation, we examined by quantitative real-time PCR the responses of transgenics expressing the WT BZR1

(E and F) BL does not induce the accumulation of BZR1$^{2K/R}$. 10-day-old transgenic *Arabidopsis* seedlings carrying $_{pro}$*BZR1::BZR1-GFP* or $_{pro}$*BZR1::BZR1*$^{2K/R}$*-GFP* were treated with a combination of 200 μM cycloheximide + 1 μM BL (E) or 2 μM BRZ (F), and total proteins extracted at indicated time points were immunoblotted with αGFP (IB: αGFP) antibody. Ponceau staining for Rubisco levels was used as a loading control.
See also Figure S3.

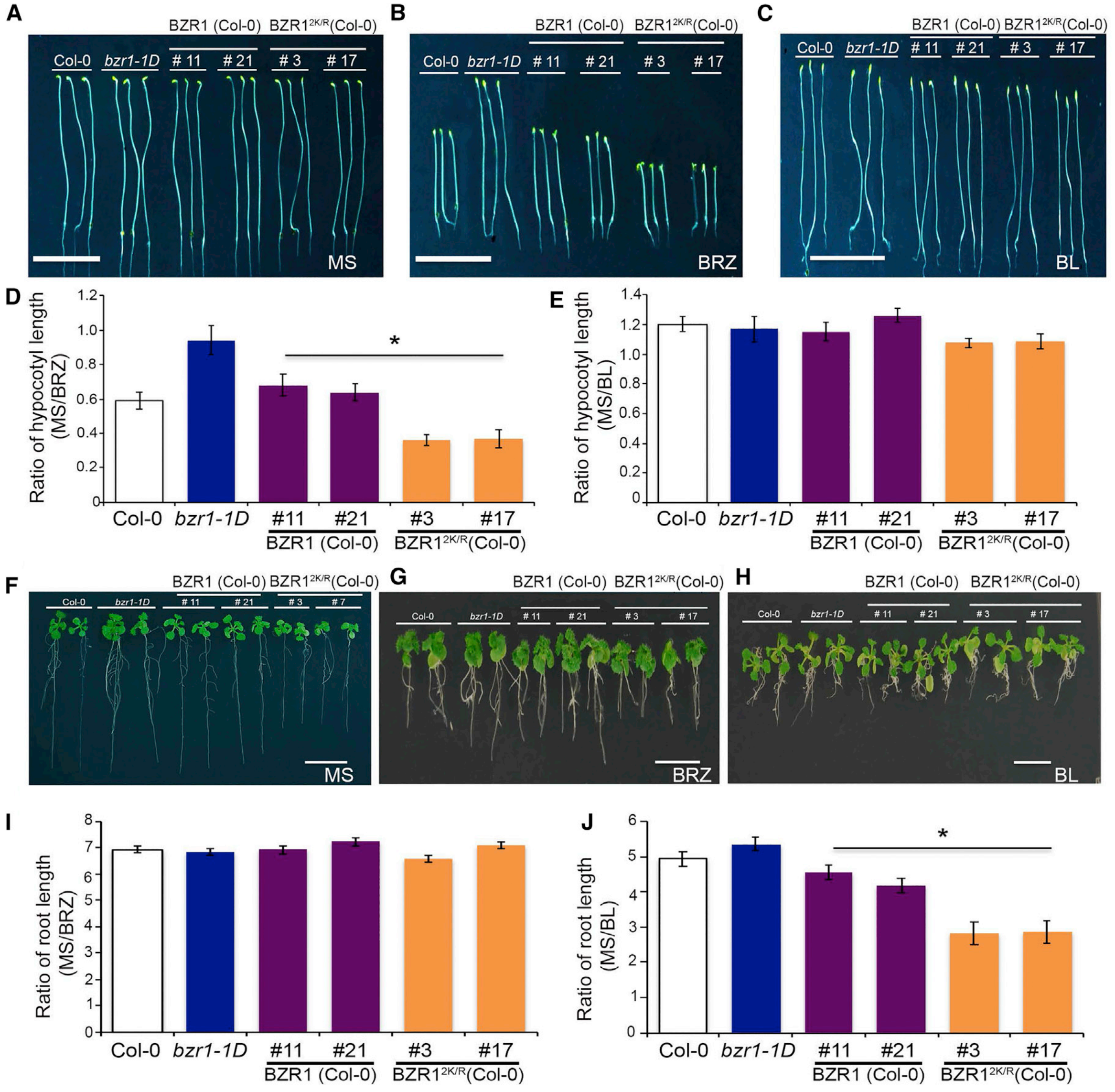

Figure 4. SUMOylated BZR1 Promotes BR-Mediated Plant Growth

(A) Representative image of hypocotyl lengths of 6-day-old seedlings of Col-0, *bzr1-1D*, transgenic $_{pro}$*BZR1::BZR1-GFP*, and transgenic $_{pro}$*BZR1::BZR1$^{2K/R}$-GFP* grown on ½ Murashige and Skoog.

(B and C) Representative image of hypocotyl lengths of 6-day-old seedlings of Col-0, *bzr1-1D*, transgenic $_{pro}$*BZR1::BZR1-GFP*, and transgenic $_{pro}$*BZR1::BZR1$^{2K/R}$-GFP* grown on BRZ (2 μM) (B) and BL (1 μM) (C) medium, respectively.

(D and E) Quantification of the ratio of hypocotyl lengths of BRZ treated (D) and BL treated (E) with reference to untreated seedlings in the dark.

(F) Representative image of root lengths of 12-day-old young adult plants of Col-0, *bzr1-1D*, transgenic $_{pro}$*BZR1::BZR1-GFP*, and transgenic $_{pro}$*BZR1::BZR1$^{2K/R}$-GFP* grown on ½ Murashige and Skoog.

(G and H) Representative image of root lengths of 12-day-old young adult plants of Col-0, *bzr1-1D*, transgenic $_{pro}$*BZR1::BZR1-GFP*, and transgenic $_{pro}$*BZR1::BZR1$^{2K/R}$-GFP* grown on BRZ (2 μM) (G) and BL (1 μM) medium (H), respectively.

(I and J) Quantification of the ratio of root growth of BRZ treated (D) and BL treated (E) with reference to untreated plants.

Scale bar, 1 cm.

Error bars indicate SE (n = 20). Asterisks indicate significant differences from Col-0. See also Figure S4.

and SUMO-deficient BZR1 under the own promoter to BRZ and BL treatment. We interrogated the transcript levels of different downstream target genes from seedlings treated with 2 μM BRZ and 1 μM of BL along with untreated seedlings. As shown in Figure S4, BL treatment of SUMO-deficient own promoter BZR1 plants is unable to efficiently regulate the transcription of BR-responsive target genes compared to Col-0 (WT) and own promoter WT BZR1 lines. These data indicate that $_{pro}$*BZR1::BZR1$^{2K/R}$-GFP* transgene is acting in a dominant manner to affect endogenous BZR1 activity.

SUMOylation of BZR1 Modulates Its Nucleocytoplasmic Distribution

To test whether SUMOylation affected BZR1 localization in transgenic plants, we analyzed native promoter-driven BZR1-GFP ($_{pro}$*BZR1::BZR1*-GFP) and non-SUMO BZR1$^{2K/R}$-GFP ($_{pro}$*BZR1::BZR1$^{2K/R}$-GFP*) localization pattern in roots treated with BL or BRZ. We treated the transgenic seedlings with BL (100 nM), and this caused an expected significant increase in the nuclear levels of WT BZR1 and a corresponding decrease in its cytoplasmic localization in the cells of the transition-elongation zone of roots at different time points (0–60 min). However, we did not observe any clear effect on the ratio of nuclear/cytoplasmic localization of non-SUMOylatable BZR1$^{2K/R}$-GFP (Figures 5A–5H), confirming our earlier transient assay findings. Quantification of the nuclear-to-cytoplasmic (N/C) ratio of BZR1-GFP showed a gradient of protein level increase along the root developmental zones, but no significant change was observed in the ratio for BZR1$^{2K/R}$-GFP (Figures 5I–5L). In contrast, following BRZ treatment (2 μM), the reduction in the nuclear localization of non-SUMO BZR1$^{2K/R}$:GFP was considerably faster than WT BZR1-GFP in all cell types but particularly in the cells of transition-elongation zone (Figures S5A–S5I). These data indicated that SUMOylation plays a key role in enabling BZR1 to locate to the plant nuclei after BL treatment.

SUMOylation of BZR1 Inhibits BIN2 Interaction

To understand the mechanistic basis of how SUMOylation of BZR1 mediates BR signaling under hormone-limiting conditions, we examined whether this posttranslational modification affected BZR1 association with its known cellular interacting partners. Because SUMOylation is critical for BZR1 stability and BZR1 abundance is known to be affected by its interaction with the GSK3-like kinase BIN2, we examined whether SUMO inhibits BIN2-BZR1 interaction. Therefore, to examine this possibility, we used Agrobacterium-mediated transient assays in *N. benthamiana* to express BZR1:GFP or BZR1$^{2K/R}$-GFP with HA-BIN2. Immunoprecipitation with anti-GFP beads to pull down BZR1 fusion proteins showed that more BIN2 is pulled down with non-SUMOylatable BZR1$^{2K/R}$-GFP (Figure 6A) compared to BZR1-GFP. To further strengthen our finding, GST pull-down assays indicated that incubating His-tagged BIN2 and GST-tagged BZR1 in the presence of the SUMO E1 and E2 that promoted SUMOylation of BZR1 reduced BIN2 interaction (Figure 6B). These data confirmed that SUMOylation of BZR1 negatively regulates its interaction with BIN2.

Salt Stress Promotes DeSUMOylation of BZR1 to Arrest Growth

Because our data indicated that SUMOylation of BZR1 plays a novel role in disrupting BZR1-BIN2 interaction, we sought to understand whether this mechanism underpinned growth control during stress.

To test this hypothesis, we ascertained the SUMOylation status of BZR1 after salt treatment using anti-SUMO1 antibodies. As expected, we observed reduced levels of SUMO-conjugated BZR1 with an increasing time frame after salt treatment with barely detectable SUMOylated BZR1 levels after 4 h of salt treatment with comparable amounts of proteins (Figure 7A), thus confirming that salt stress causes deSUMOylation of BZR1. We also monitored the level of ULP1a protein in response to salt treatment. We found that the levels of ULP1a protein increased after 2 h of salt treatment. Taken together with the evidence that ULP1a interacts with BZR1, our data indicate that accumulation of ULP1a SUMO protease promotes the deSUMOylation of BZR1 to suppress BR signaling during salt stress (Figures 7B and 7C).

To gain more insight into the role of BZR1 SUMOylation and its role during abiotic stress, we then extended our study to analyze root growth in presence of salt of 12-day-old transgenic lines of own promoter BZR1-GFP ($_{pro}$*BZR1::BZR1*-GFP) and non-SUMOylatable BZR1$^{2K/R}$-GFP ($_{pro}$*BZR1::BZR1$^{2K/R}$*-GFP) and compared them to Col-0 and the dominant positive mutant *bzr1-1D*. We did not notice any significant difference in root growth inhibition among the genotypes in salt, but the non-SUMOylatable BZR1$^{2K/R}$-GFP lines did exhibit reduced root growth in Murashige and Skoog media without salt (Figures 7D and 7E). This could probably be because WT BZR1 is deSUMOylated in the presence of salt, as would be the case in *BZR1$^{2K/R}$-GFP* lines, and hence exhibiting similar reduced root growth phenotypes. Recently, application of exogenous BRs was shown to aid plants to recover growth after salt stress [41, 42]. We next grew seedlings of the various genotypes on 100 mM NaCl for 6 days and then transferred them to plates with BL for another 6 days. Transgenic WT *BZR1-GFP* seedlings showed an increase in root length similar to Col-0 and *bzr1-1D*; however, seedlings expressing the non-SUMOylatable *BZR1$^{2K/R}$-GFP* failed to show any significant recovery of root growth (Figure 7F), indicating that, even in the presence of BL, recovery of growth after stress is SUMO dependent. Quantification of the fresh weights of the plants in different treatments also confirmed the above observation (Figure S6). Furthermore, colocalization experiments with BZR1-GFP and ULP1a-mCherry transiently co-expressed in *N. benthamiana* showed BZR1 and ULP1a colocalized after salt treatment (Figure 7G). These data support our previous finding that ULP1a accumulates in the cytoplasm during salt stress. Taken together, we conclude that the increased abundance of ULP1a in the cytoplasm causes deSUMOylation of BZR1, affecting its stability and preventing its nuclear import, leading to growth repression during salt stress.

DISCUSSION

Brassinosteroids are steroid hormones required for the regulation of a variety of physiological processes from cellular expansion and proliferation to developmental programs, leading to morphogenesis. BR signaling is dependent on a series of phosphorylation events to modulate the function of BZR1/BES1 transcription factors that regulate the expression of BR-responsive genes. In this study, we demonstrate that SUMO conjugation to BZR1 provides a new mechanism to regulate BR signaling to tailor plant growth and development to its environment. Our data demonstrate that SUMOylation inhibits interaction between BZR1 and the kinase, BIN2. Phosphorylation by BIN2 is critical for BZR1 degradation and BR signaling [18, 19, 40]. Non-SUMOylatable BZR1 has greater retention in the cytoplasm, interacts more with BIN2, and is less stable; therefore, our data indicate that the major effect of SUMOylation is to prevent BZR1 from being a substrate for BIN2 kinase. Given the

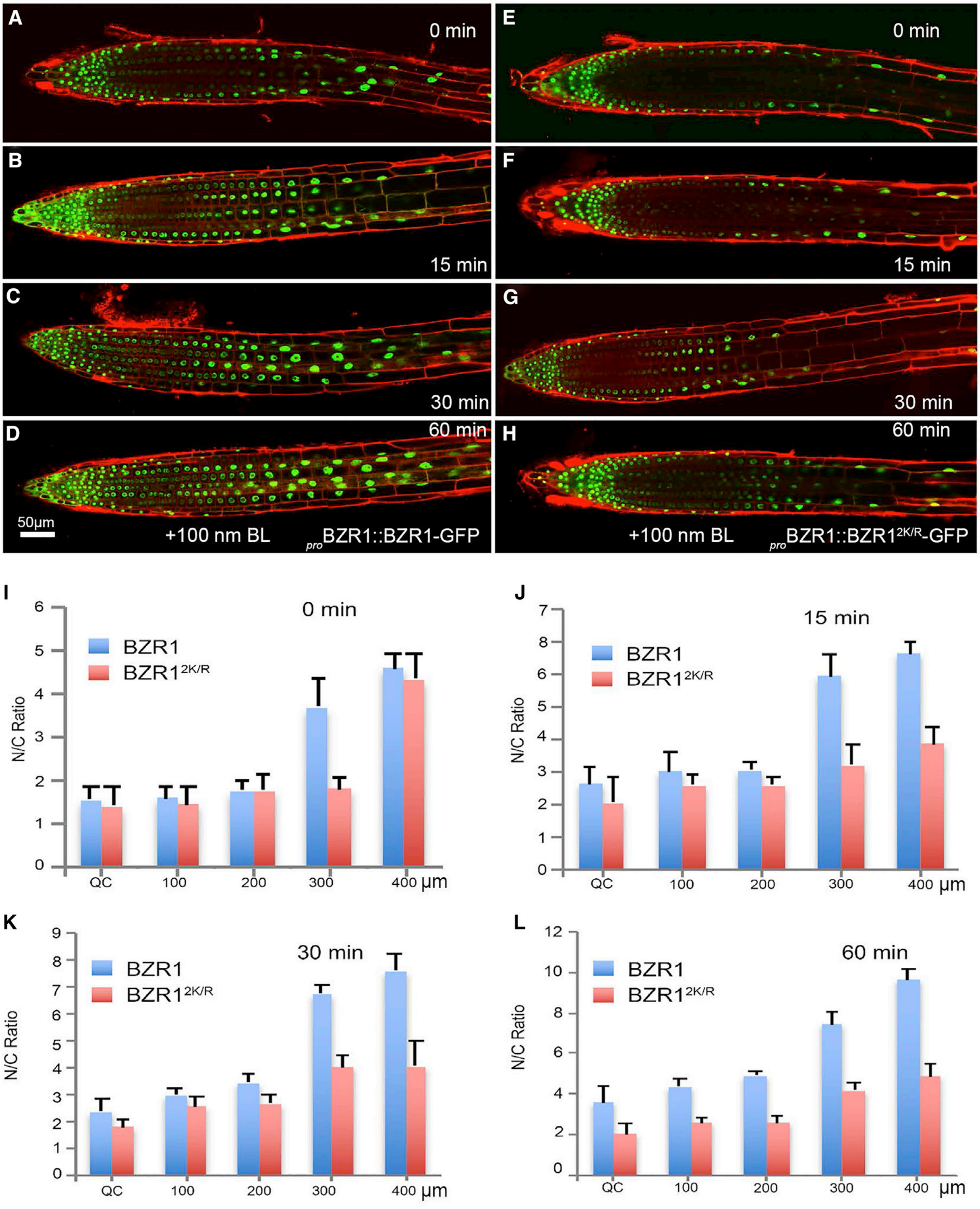

Figure 5. BL-Induced Nuclear Import of BZR1 Is Enhanced by SUMO

(A–D) Confocal images of 4-day-old transgenic $_{pro}$*BZR1::BZR1-GFP* seedlings at 0 (A), 15 (B), 30 (C), and 60 (D) min of treatment with 100 nM BL.

(E–H) Confocal images of 4-day-old transgenic $_{pro}$*BZR1::BZR1$^{2K/R}$-GFP* seedlings at 0 (E), 15 (F), 30 (G), and 60 (H) min of treatment with 100 nM BL.

(I–L) Nuclear/cytoplasm (N/C) ratios of the GFP signals from root tip region to elongation zone of the roots at 0- (I), 15- (J), 30- (K), and 60-min (L) time points after treated with 100 nM BL.

Error bars indicate SE (n = 20). Asterisks indicate significant differences from Col-0. See also Figure S5.

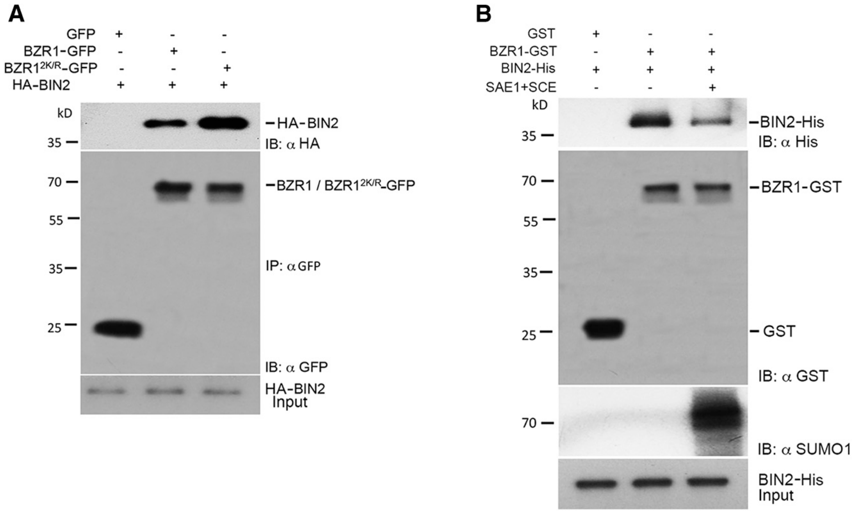

Figure 6. SUMOylation of BZR1 Interferes with Its Interaction with BIN2

(A) BZR1$^{2K/R}$-GFP interacts strongly with BIN2 compared to BZR1-GFP. *N. benthamiana* leaves transiently expressing BZR1-GFP or BZR1$^{2K/R}$-GFP with HA-BIN2 were collected for immunoprecipitation. Subsequently, total protein was subjected to immunoprecipitation with αGFP immunoaffinity beads (IP: αGFP) followed by immunoblot analysis with αHA (IB: αHA) antibodies to detect HA-BIN2 and αGFP (IB: αGFP) antibodies to detect BZR1-GFP. GFP was used as a negative control.

(B) SUMO inhibits the interaction of BZR1-BIN2. BZR1-GST mixed with and without His-SUMO1 was incubated with His-BIN2 and pulled down with BZR1-GST. The eluates were probed with αHis to detect His-BIN2 or αGST to detect BZR1-GST or antibodies or αSUMO1 to detect the SUMO levels of BZR1.

importance of BZR1 and the conservation of the identified SUMO sites across plant species, our data place SUMO at the core of BR signaling. An intriguing observation of generating non-SUMO forms of BZR1 (BZR1$^{2K/R}$) is the dominant effects of BZR1$^{2K/R}$ on seedling growth when expressed in the genetic background that contains a WT BZR1. It has been recently reported that phosphorylated BZR1 has a higher tendency to dimerize with non-phosphorylated BZR1 and thus can trap the non-phosphorylated BZR1 to form a dimer [16] for co-degradation. Because the non-SUMO BZR1$^{2K/R}$ has a higher tendency to interact with the BIN2 kinase, we postulate that it traps the endogenous WT BZR1 by dimerization, thus affecting WT BZR1 activity, resulting in altered levels of gene expression and the short hypocotyl phenotype for $_{pro}$*BZR1::*BZR1$^{2K/R}$-GFP transgenic lines in the Col-0 background. Our data also establish a critical effect of salt stress on BR signaling through BZR1 SUMOylation. Upon salt stress, BZR1 is rapidly deSUMOylated, therefore allowing greater interaction with BIN2 and consequently faster degradation of BZR1 to inhibit growth. This effect is even more pronounced when seedlings are transferred from salt-containing plates to plates supplemented with exogenous BL that should stimulate growth. However, plants expressing non-SUMO BZR1$^{2K/R}$ are less able to stimulate growth than WT Col-0, indicating that SUMOylation of BZR1 is a prerequisite for growth, even in the presence of brassinosteroids. The data therefore reveal a mechanism whereby environmental cues, such as salt stress, can impinge on BR-mediated growth through SUMO status of BZR1. In addition, we also discover the significance of SUMO protease that regulates the nucleocytoplasmic localization through deconjugation of SUMO from its target protein BZR1. DeSUMOylation of BZR1 abolishes its ability to translocate from cytoplasm to nucleus (Figure 5).

Plant deSUMOylating proteases belonging to the ULP gene family, extensively studied in *Arabidopsis*, are assumed to contain eight ULPs. It is most likely that specificity is imparted by deSUMOylating enzymes rather than by SUMO-conjugating enzymes, especially after the discovery of a new class of SUMO protease family, Desi SUMO protease in plants [33]. As the ULPs are involved in stress resistance, they could provide a possible target for the generation of high-yielding, stress-resistant crops. This raises an interesting concern in regards to their specificity within the SUMO system. Furthermore, our data identify ULP1a SUMO protease as a new component of BR signaling pathway. We found that SUMO protease ULP1a is localized in the cytoplasm, where it targets BZR1 for deSUMOylation and thereby acts as a negative regulator of BR signaling (Figures 1 and 2). Salt stress induces the accumulation of ULP1a and therefore stimulates the deSUMOylation of BZR1 although BR signaling triggers ULP1a degradation and promotes BZR1 SUMOylation (Figure 7H). Our evidence places ULP1a as the conduit to environmental regulation of BR signaling in plants.

Plant SUMO proteases are emerging as key components of multiple signaling pathways [27, 33, 34, 43, 44]. The expansion of the SUMO protease gene family rather than the SUMO E3 ligase family in plants has given rise to the notion that the specificity within the SUMO system arises from the deconjugation part of the SUMO cycle. Nevertheless, thousands of SUMO substrates have been identified with only tens of SUMO proteases annotated in plant genomes; therefore, it is likely that each SUMO protease targets multiple substrates.

We have recently identified a number of targets for different SUMO proteases that occur in different subcellular localizations. Overlay tolerant to Salt1/2 (OTS1/2) is localized in the nucleus and is known to target DELLA proteins to regulate plant growth through a (gibberellic acid) GA-independent mechanism [40]. OTS SUMO proteases are also known to modulate salicylic acid (SA)- and jasmonic acid (JA)-mediated defense response in *Arabidopsis* [34, 45]. Recently, we have shown the significance of OTS1/2 in hydropatterning and lateral root adaptive responses in plants [26]. We have also identified a novel class of cytoplasmic and membrane-localized SUMO proteases known as Desi SUMO proteases [33]. A member of this SUMO protease Desi3a plays a significant role in targeting the bacterial immune receptor, FLS2, to suppress inappropriate immune responses, which has implications for BR signaling through the BAK1 co-receptor. This evidence indicates that the subcellular localization of these SUMO proteases may have a critical role in its substrate selectivity.

Our data suggest that, to fully realize the impact of the SUMO system in plants, it will be important to understand the

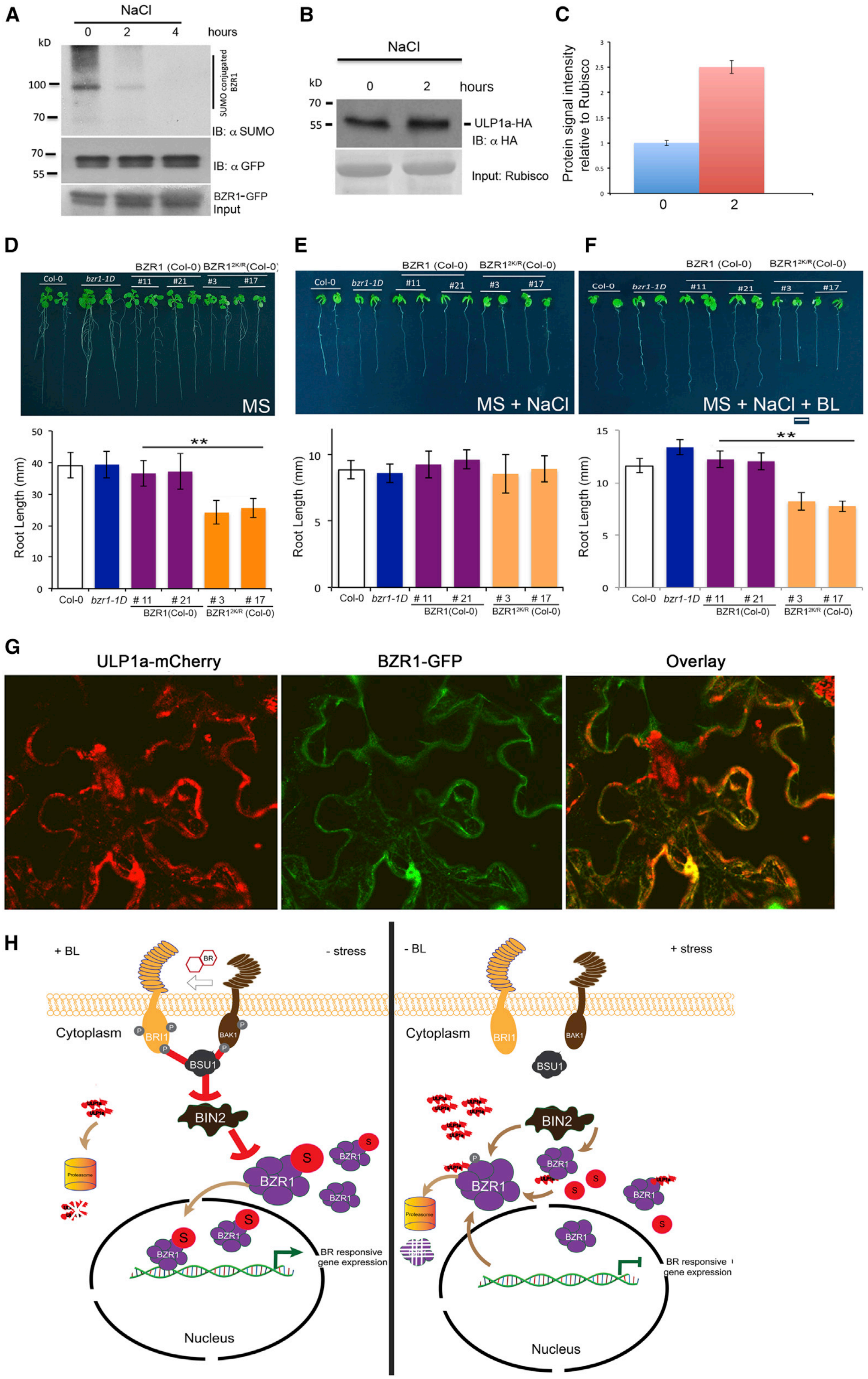

(legend on next page)

intersections of the core SUMO system with their cognate targets, such as in the case for ULP1a and BZR1. Furthermore, generating non-SUMO versions of target substrates should yield new insight into molecular pathways that may be utilized in the future to boost crop productivity under stress in a changing climate.

STAR★METHODS

Detailed methods are provided in the online version of this paper and include the following:

- KEY RESOURCES TABLE
- LEAD CONTACT AND MATERIALS AVAILABILITY
- EXPERIMENTAL MODEL AND SUBJECT DETAILS
- METHOD DETAILS
  - Plant Materials and Growth Conditions
  - Plasmid Construction and Plant Transformation
  - Site Directed Mutagenesis
  - Seedling Growth Inhibition Assay
  - Total RNA Extraction and Quantitative RT-PCR
  - Immunoprecipitation and Co-immunoprecipitation Assay
  - Protein Extraction and Western Blot Analysis
  - Confocal Microscopy, Quantification of Fluorescence Signal and Imaging
- QUANTIFICATION AND STATISTICAL ANALYSIS
- DATA AND CODE AVAILABILITY

SUPPLEMENTAL INFORMATION

Supplemental Information can be found online at https://doi.org/10.1016/j.cub.2020.01.089.

ACKNOWLEDGMENTS

Authors would like to thank Dr. Tim Hawkins and Ms. Joanne Robson for assistance with confocal microscopy. This manuscript is dedicated to the memory of Mrs. Kuntala Chakraborty. The research is financially supported by the European Research Council grant (SUMOrice grant no. 310235) and Biological and Biotechnological Research Council grant (Hydropatterning grant reference; BB/M002136/1) to A.S.

AUTHOR CONTRIBUTIONS

Conceptualization, M.S. and A.S.; Methodology, M.S., A.K.S., and A.S.; Investigation, M.S., A.K.S., A.C., B.O.-P., and C.Z.; Writing – Original Draft, M.S., A.K.S., and A.S.; Writing – Review & Editing, M.S. and A.S.; Funding Acquisition, A.S.; Resources, C.Z.

DECLARATION OF INTERESTS

The authors declare no competing interests.

Received: June 28, 2019
Revised: January 7, 2020
Accepted: January 30, 2020
Published: February 27, 2020

REFERENCES

1. Depuydt, S., and Hardtke, C.S. (2011). Hormone signalling crosstalk in plant growth regulation. Curr. Biol. *21*, R365–R373.
2. Santner, A., and Estelle, M. (2009). Recent advances and emerging trends in plant hormone signalling. Nature *459*, 1071–1078.
3. Clouse, S.D. (2011). Brassinosteroid signal transduction: from receptor kinase activation to transcriptional networks regulating plant development. Plant Cell *23*, 1219–1230.
4. Wang, X., and Chory, J. (2006). Brassinosteroids regulate dissociation of BKI1, a negative regulator of BRI1 signaling, from the plasma membrane. Science *313*, 1118–1122.
5. Li, J., and Chory, J. (1997). A putative leucine-rich repeat receptor kinase involved in brassinosteroid signal transduction. Cell *90*, 929–938.
6. Gou, X., Yin, H., He, K., Du, J., Yi, J., Xu, S., Lin, H., Clouse, S.D., and Li, J. (2012). Genetic evidence for an indispensable role of somatic embryogenesis receptor kinases in brassinosteroid signaling. PLoS Genet. *8*, e1002452.

Figure 7. Salt Stress Promotes DeSUMOylation of BZR1 to Arrest Growth

(A) NaCl inhibits SUMOylation of BZR1. Immunoprecipitation (IP: αGFP) experiments were carried out with αGFP beads from total proteins derived from transgenic lines expressing $_{pro}$*BZR1::BZR1-GFP* in Col-0 background after treatment with 100 mM NaCl at time points of 0, 2, and 4 h. Immunoblots were probed with αGFP (IB: αGFP) or αSUMO1/2 (IB: αSUMO1) antibodies.

(B) NaCl causes accumulation of ULP1a protein. 10-day-old *Arabidopsis* seedlings expressing *35S::ULP1a-HA* were treated with a combination of 200 μM cycloheximide and 100 mM NaCl, and total proteins extracted at indicated time points were immunoblotted with αHA (IB: αHA) antibody. Ponceau staining for Rubisco was used to ascertain total protein loading in each lane.

(C) Quantification of (B) using ImageJ software.

(D) Representative image and quantification of root lengths of 12-day-old young adult plants of Col-0, *bzr1-1D*, transgenic $_{pro}$*BZR1::BZR1-GFP*, and transgenic $_{pro}$*BZR1::BZR1*$^{2K/R}$*-GFP* grown on ½ Murashige and Skoog. Scale bar, 1 cm.

(E) Representative image and quantification of root lengths of 12-day-old young adult plants of Col-0, *bzr1-1D*, transgenic $_{pro}$*BZR1:BZR1-GFP*, and transgenic $_{pro}$*BZR1:BZR1*$^{2K/R}$*-GFP* grown on ½ Murashige and Skoog with 100 mM NaCl. Scale bar, 1 cm.

(F) Representative image and quantification of root lengths of 12-day-old young adult plants of Col-0, *bzr1-1D*, transgenic $_{pro}$*BZR1::BZR1-GFP*, and transgenic $_{pro}$*BZR1::BZR1*$^{2K/R}$*-GFP* grown on ½ Murashige and Skoog with NaCl for 6 days and were then moved to ½ Murashige and Skoog with 1 μM BL for the next 6 days. Scale bar, 1 cm.

(G) BZR1 and ULP1a colocalize in the cytoplasm during salt stress. *N. benthamiana* leaves co-infiltrated with Agrobacterium carrying ULP1a-mCherry and BZR1-GFP were analyzed for fluorescence after 3 days. Before imaging, the leaves were infiltrated with 100 mM NaCl and incubated for 2 h to see the effect of the treatments. Images were obtained using confocal laser scanning microscope Carl Zeiss Airyscan 880. Scale bar, 20 μm.

(H) A model for the influence of SUMO on BR signaling. BZR1 gets deSUMOylated by ULP1a in response to abiotic stress, hence preventing its nuclear import and disrupting the signaling pathway. In absence of stress and availability of BL, BZR1 is SUMOylated as ULP1a does not accumulate. The SUMOylated BZR1 has reduced interaction with BIN2, which itself gets inhibited by the upstream BL signaling components, and hence, BZR1 moves into the nucleus and promotes the expression of BR-responsive genes, whereas during salt stress, ULP1a accumulates, causing deSUMOylation of BZR1 to cause increased interaction with BIN2 and hence phosphorylation and subsequent degradation of BZR1. As a result of deSUMOylation, the nuclear import of BZR1 is hindered, thus inhibiting the expression of BR-responsive genes.

Error bars indicate SE (n = 20). Asterisks indicate significant differences from Col-0. See also Figure S6.

7. She, J., Han, Z., Kim, T.W., Wang, J., Cheng, W., Chang, J., Shi, S., Wang, J., Yang, M., Wang, Z.Y., and Chai, J. (2011). Structural insight into brassinosteroid perception by BRI1. Nature *474*, 472–476.
8. Hothorn, M., Belkhadir, Y., Dreux, M., Dabi, T., Noel, J.P., Wilson, I.A., and Chory, J. (2011). Structural basis of steroid hormone perception by the receptor kinase BRI1. Nature *474*, 467–471.
9. Kinoshita, T., Caño-Delgado, A., Seto, H., Hiranuma, S., Fujioka, S., Yoshida, S., and Chory, J. (2005). Binding of brassinosteroids to the extracellular domain of plant receptor kinase BRI1. Nature *433*, 167–171.
10. Li, J., Wen, J., Lease, K.A., Doke, J.T., Tax, F.E., and Walker, J.C. (2002). BAK1, an Arabidopsis LRR receptor-like protein kinase, interacts with BRI1 and modulates brassinosteroid signaling. Cell *110*, 213–222.
11. Nam, K.H., and Li, J. (2002). BRI1/BAK1, a receptor kinase pair mediating brassinosteroid signaling. Cell *110*, 203–212.
12. Kim, T.W., Guan, S., Burlingame, A.L., and Wang, Z.Y. (2011). The CDG1 kinase mediates brassinosteroid signal transduction from BRI1 receptor kinase to BSU1 phosphatase and GSK3-like kinase BIN2. Mol. Cell *43*, 561–571.
13. Kim, T.W., Guan, S., Sun, Y., Deng, Z., Tang, W., Shang, J.X., Sun, Y., Burlingame, A.L., and Wang, Z.Y. (2009). Brassinosteroid signal transduction from cell-surface receptor kinases to nuclear transcription factors. Nat. Cell Biol. *11*, 1254–1260.
14. Mora-García, S., Vert, G., Yin, Y., Caño-Delgado, A., Cheong, H., and Chory, J. (2004). Nuclear protein phosphatases with Kelch-repeat domains modulate the response to brassinosteroids in Arabidopsis. Genes Dev. *18*, 448–460.
15. Kim, Y.M., Seo, Y.H., Park, C.B., Yoon, S.H., and Yoon, G. (2010). Roles of GSK3 in metabolic shift toward abnormal anabolism in cell senescence. Ann. N Y Acad. Sci. *1201*, 65–71.
16. Kim, B., Jeong, Y.J., Corvalán, C., Fujioka, S., Cho, S., Park, T., and Choe, S. (2014). Darkness and gulliver2/phyB mutation decrease the abundance of phosphorylated BZR1 to activate brassinosteroid signaling in Arabidopsis. Plant J. *77*, 737–747.
17. Bai, M.Y., Zhang, L.Y., Gampala, S.S., Zhu, S.W., Song, W.Y., Chong, K., and Wang, Z.Y. (2007). Functions of OsBZR1 and 14-3-3 proteins in brassinosteroid signaling in rice. Proc. Natl. Acad. Sci. USA *104*, 13839–13844.
18. Gampala, S.S., Kim, T.W., He, J.X., Tang, W., Deng, Z., Bai, M.Y., Guan, S., Lalonde, S., Sun, Y., Gendron, J.M., et al. (2007). An essential role for 14-3-3 proteins in brassinosteroid signal transduction in Arabidopsis. Dev. Cell *13*, 177–189.
19. Vert, G., and Chory, J. (2006). Downstream nuclear events in brassinosteroid signalling. Nature *441*, 96–100.
20. Peng, P., Yan, Z., Zhu, Y., and Li, J. (2008). Regulation of the Arabidopsis GSK3-like kinase BRASSINOSTEROID-INSENSITIVE 2 through proteasome-mediated protein degradation. Mol. Plant *1*, 338–346.
21. Tang, W., Yuan, M., Wang, R., Yang, Y., Wang, C., Oses-Prieto, J.A., Kim, T.W., Zhou, H.W., Deng, Z., Gampala, S.S., et al. (2011). PP2A activates brassinosteroid-responsive gene expression and plant growth by dephosphorylating BZR1. Nat. Cell Biol. *13*, 124–131.
22. Yu, X., Li, L., Zola, J., Aluru, M., Ye, H., Foudree, A., Guo, H., Anderson, S., Aluru, S., Liu, P., et al. (2011). A brassinosteroid transcriptional network revealed by genome-wide identification of BESI target genes in Arabidopsis thaliana. Plant J. *65*, 634–646.
23. Sun, Y., Fan, X.Y., Cao, D.M., Tang, W., He, K., Zhu, J.Y., He, J.X., Bai, M.Y., Zhu, S., Oh, E., et al. (2010). Integration of brassinosteroid signal transduction with the transcription network for plant growth regulation in Arabidopsis. Dev. Cell *19*, 765–777.
24. He, J.X., Gendron, J.M., Sun, Y., Gampala, S.S., Gendron, N., Sun, C.Q., and Wang, Z.Y. (2005). BZR1 is a transcriptional repressor with dual roles in brassinosteroid homeostasis and growth responses. Science *307*, 1634–1638.
25. Yin, Y., Vafeados, D., Tao, Y., Yoshida, S., Asami, T., and Chory, J. (2005). A new class of transcription factors mediates brassinosteroid-regulated gene expression in Arabidopsis. Cell *120*, 249–259.
26. Orosa-Puente, B., Leftley, N., von Wangenheim, D., Banda, J., Srivastava, A.K., Hill, K., Truskina, J., Bhosale, R., Morris, E., Srivastava, M., et al. (2018). Root branching toward water involves posttranslational modification of transcription factor ARF7. Science *362*, 1407–1410.
27. Conti, L., Price, G., O'Donnell, E., Schwessinger, B., Dominy, P., and Sadanandom, A. (2008). Small ubiquitin-like modifier proteases OVERLY TOLERANT TO SALT1 and -2 regulate salt stress responses in Arabidopsis. Plant Cell *20*, 2894–2908.
28. Capili, A.D., and Lima, C.D. (2007). Structure and analysis of a complex between SUMO and Ubc9 illustrates features of a conserved E2-Ubl interaction. J. Mol. Biol. *369*, 608–618.
29. Kerscher, O., Felberbaum, R., and Hochstrasser, M. (2006). Modification of proteins by ubiquitin and ubiquitin-like proteins. Annu. Rev. Cell Dev. Biol. *22*, 159–180.
30. Jentsch, S., and Pyrowolakis, G. (2000). Ubiquitin and its kin: how close are the family ties? Trends Cell Biol. *10*, 335–342.
31. Verger, A., Perdomo, J., and Crossley, M. (2003). Modification with SUMO. A role in transcriptional regulation. EMBO Rep. *4*, 137–142.
32. Hay, R.T. (2001). Protein modification by SUMO. Trends Biochem. Sci. *26*, 332–333.
33. Khan, M., Rozhon, W., Unterholzner, S.J., Chen, T., Eremina, M., Wurzinger, B., Bachmair, A., Teige, M., Sieberer, T., Isono, E., and Poppenberger, B. (2014). Interplay between phosphorylation and SUMOylation events determines CESTA protein fate in brassinosteroid signalling. Nat. Commun. *5*, 4687.
34. Orosa, B., Yates, G., Verma, V., Srivastava, A.K., Srivastava, M., Campanaro, A., De Vega, D., Fernandes, A., Zhang, C., Lee, J., et al. (2018). SUMO conjugation to the pattern recognition receptor FLS2 triggers intracellular signalling in plant innate immunity. Nat. Commun. *9*, 5185.
35. Srivastava, A.K., Orosa, B., Singh, P., Cummins, I., Walsh, C., Zhang, C., Grant, M., Roberts, M.R., Anand, G.S., Fitches, E., and Sadanandom, A. (2018). SUMO suppresses the activity of the jasmonic acid receptor CORONATINE INSENSITIVE1. Plant Cell *30*, 2099–2115.
36. Hermkes, R., Fu, Y.F., Nürrenberg, K., Budhiraja, R., Schmelzer, E., Elrouby, N., Dohmen, R.J., Bachmair, A., and Coupland, G. (2011). Distinct roles for Arabidopsis SUMO protease ESD4 and its closest homolog ELS1. Planta *233*, 63–73.
37. Tao, J.J., Chen, H.W., Ma, B., Zhang, W.K., Chen, S.Y., and Zhang, J.S. (2015). The role of ethylene in plants under salinity stress. Front. Plant Sci. *6*, 1059.
38. Zhu, T., Deng, X., Zhou, X., Zhu, L., Zou, L., Li, P., Zhang, D., and Lin, H. (2016). Ethylene and hydrogen peroxide are involved in brassinosteroid-induced salt tolerance in tomato. Sci. Rep. *6*, 35392.
39. Wang, Z.Y., Nakano, T., Gendron, J., He, J., Chen, M., Vafeados, D., Yang, Y., Fujioka, S., Yoshida, S., Asami, T., and Chory, J. (2002). Nuclear-localized BZR1 mediates brassinosteroid-induced growth and feedback suppression of brassinosteroid biosynthesis. Dev. Cell *2*, 505–513.
40. Hickey, C.M., Wilson, N.R., and Hochstrasser, M. (2012). Function and regulation of SUMO proteases. Nat. Rev. Mol. Cell Biol. *13*, 755–766.
41. Ryu, H., Kim, K., Cho, H., Park, J., Choe, S., and Hwang, I. (2007). Nucleocytoplasmic shuttling of BZR1 mediated by phosphorylation is essential in Arabidopsis brassinosteroid signaling. Plant Cell *19*, 2749–2762.
42. Conti, L., Nelis, S., Zhang, C., Woodcock, A., Swarup, R., Galbiati, M., Tonelli, C., Napier, R., Hedden, P., Bennett, M., and Sadanandom, A. (2014). Small Ubiquitin-like Modifier protein SUMO enables plants to control growth independently of the phytohormone gibberellin. Dev. Cell *28*, 102–110.
43. Geng, Y., Wu, R., Wee, C.W., Xie, F., Wei, X., Chan, P.M., Tham, C., Duan, L., and Dinneny, J.R. (2013). A spatio-temporal understanding of growth

regulation during the salt stress response in Arabidopsis. Plant Cell *25*, 2132–2154.

44. He, J.X., Gendron, J.M., Yang, Y., Li, J., and Wang, Z.Y. (2002). The GSK3-like kinase BIN2 phosphorylates and destabilizes BZR1, a positive regulator of the brassinosteroid signaling pathway in Arabidopsis. Proc. Natl. Acad. Sci. USA *99*, 10185–10190.

45. Bailey, M., Srivastava, A., Conti, L., Nelis, S., Zhang, C., Florance, H., Love, A., Milner, J., Napier, R., Grant, M., and Sadanandom, A. (2016). Stability of small ubiquitin-like modifier (SUMO) proteases OVERLY TOLERANT TO SALT1 and -2 modulates salicylic acid signalling and SUMO1/2 conjugation in Arabidopsis thaliana. J. Exp. Bot. *67*, 353–363.

STAR★METHODS

KEY RESOURCES TABLE

REAGENT or RESOURCES	SOURCE	IDENTIFIER
Antibodies		
GFP-Trap	Chromotek	Cat# GFP-Trap-MA
Rabbit Polyclonal anti-SUMO1 antibody	[27]	N/A
Rabbit Monoclonal anti-GFP antibody	Abcam	Cat#Ab6556; RRID: AB_305564
Monoclonal anti-HA antibody	Roche	Cat#118674223001; RRID: AB_390918
Mouse Monoclonal anti-His antibody	Sigma Aldrich	Cat#H1029; RRID: AB_2687993
Rat Monoclonal anti-GST antibody	Sigma Aldrich	Cat#SAB4200055; RRID: AB_10603625
Goat anti-rabbit IgG-HRP secondary antibody	Sigma-Aldrich	Cat#A0545; RRID: AB_2279879
Goat anti-mouse IgG-HRP secondary antibody	Sigma-Aldrich	Cat#A9917; RRID: AB_954556
Rabbit anti-rat IgG-HRP secondary antibody	Sigma-Aldrich	Cat#A5795; RRID: AB_956022
Bacterial Strains		
Escherichia coli DH5α	NEB	Cat #C2987K
Escherichia coli BL 21	NEB	Cat #C2530H
Agrobacterium tumefaciens (strain GV3101)	N/A	N/A
Chemicals		
Murashige and Skoog Basal Medium	Duchefa	Cat#M0233
Agar	Melford	Cat#A20250
LB-Agar	Melford	Cat#L24033
BASTA	Sigma-Aldrich	Cat#45520
IPTG	Sigma-Aldrich	Cat#I6758
Glutathione Sepharose 4B	GE Healthcare	17-0969-01
Cycloheximide	Sigma-Aldrich	Cat#C4859
RNeasy Kit	QIAGEN	Cat#74904
Ponceau S	Sigma-Aldrich	Cat#P7170-1L
Acrylamide/Bis-acrylamide, 30% solution	Sigma-Aldrich	Cat#A3699
Ammonium persulfate	Sigma-Aldrich	Cat#A3678
N,N,N,N-Tetramethylethylenediamine	Sigma-Aldrich	Cat#T9281
Magnesium chloride hexahydrate	Merck	Cat#105833
Glycerol	Merck	Cat#104098
cOmplete, EDTA-free Protease Inhibitor Cocktail	Roche	Cat#4693132001
2-mercaptoethanol	Sigma-Aldrich	Cat#M3148
DTT	Malford	Cat#D11000
Critical Commercial Assay		
RNeasy Plant Mini Kit	QIAGEN	Cat#74904
SYBR Green	Sigma-Aldrich	
SuperScript III Reverse Transcriptase	Thermo Fisher	Cat#18080093
Zymoclean Gel DNA Recovery	Zymo research	Cat#D4007
ZymoPURE II Plasmid Kits	Zymo research	Cat#D4201
Experimental Models		
Arabidopsis ots1 ots2	[27]	N/A
Arabidopsis ulp1a	This Study	N/A
Arabidopsis bzr1-1D	This Study	N/A
Arabidopsis 35S::BZR1-GFP	This Study	N/A
Arabidopsis 35S::BZR1K280/320R-GFP	This Study	N/A

(*Continued on next page*)

Continued		
REAGENT or RESOURCES	SOURCE	IDENTIFIER
Arabidopsis proBZR1::BZR1-GFP	This Study	N/A
Arabidopsis proBZR1::BZR1K280/320R-GFP	This Study	N/A
Arabidopsis 35S::ULP1-HA	This Study	N/A
Oligonucleotides		
Primers are listed in Table S1	This Study	N/A
Constructs		
Constructs used are listed in Table S2		
Software and Algorithms		
ImageJ	NIH, USA	http://rsb.info.nih.gov/ij
GraphPad Prism6	Graph Pad Software	https://www.graphpad.com

LEAD CONTACT AND MATERIALS AVAILABILITY

Prof. Ari Sadanandom, Department of Biosciences, Durham University DH1 3LE UK
Phone: +44 (0) 191 33 41263
E-mail: ari.sadanandom@durham.ac.uk

This study did not generate new unique reagents. The transgenics generated in the study are available from the lead contact on request.

EXPERIMENTAL MODEL AND SUBJECT DETAILS

The wild-type *Arabidopsis* ecotype used in this study was Columbia-0 (Col). All the mutants and transgenic plants were in the Col-0 background. Adult plants were grown in soil under long day condition (16 hr light/8 hr night) at 22°C.

METHOD DETAILS

Plant Materials and Growth Conditions

Arabidopsis thaliana ecotype Columbia (Col-0) was used as the wild-type control plants. *Arabidopsis* plants used in this study were grown in environmentally controlled growth chambers as one plant per pot at 20–21°C with a 16h photoperiod for generating and progressing transgenics. For growing on plates, Murashige and Skoog (MS) medium (Duchefa) with 1% agar was used and plants were grown at 22°C with a 16h photoperiod. The T-DNA SALK lines were obtained from the Nottingham *Arabidopsis* Stock Centre. The mutant lines used in this study are: *bzr1-1D ulp1a* (SAIL_318_C01).

Plasmid Construction and Plant Transformation

All the constructs were generated by GATEWAY Cloning Technology. To generate the *BZR1*-GFP, HA-*BIN2*, HA-*ULP1a* and *ULP1a*-mCherry constructs, the corresponding cDNA fragments were PCR-amplified and cloned into pENTR D-TOPO vector. By recombination all genes were moved to their final vector indicated in Table S2. For generating $_{pro}$*BZR1::BZR1-GFP*, BZR1 promoter has been fused with CDS of *BZR1* gene and cloned in pMDC107 vector (Table S2). To generate transgenic plants Col-0 plants were transformed with either $_{pro}$*BZR1::BZR1-GFP*, or $_{pro}$*BZR1::BZR1*$^{2K/R}$*-GFP*, constructs using *Agrobacterium*-mediated floral dip method. The primers used to generate the transgenics are listed in Table S1.

Site Directed Mutagenesis

Mutated versions of BZR1 was generated by site-directed mutagenesis using the pENTR/D-TOPO clones as template. Oligonucleotide primers used to introduce the mutations are listed in Table S1. The introduction of mutations was confirmed by sequencing, performed both before and after introduction of the mutated BZR1 coding sequences into pMDC107 destination vectors using LR Clonase (Invitrogen).

Seedling Growth Inhibition Assay

Col-0, *bzr1-1D*, $_{pro}$*BZR1::BZR1-GFP* and $_{pro}$*BZR1::BZR1*$^{2K/R}$*-GFP* seeds were surface sterilized and were plated on MS plates with or without BL or BRZ and grown for 6 days in light or in dark after which the hypocotyl lengths were measured. Four-day-old seedlings of Col-0, *bzr1-1D*, $_{pro}$*BZR1::BZR1-GFP* and $_{pro}$*BZR1::BZR1*$^{2K/R}$*-GFP* were transferred to MS plates with or without BL or BRZ and grown for additional eight days before root length inhibition was measured. Col-0, *bzr1-1D*, seedlings were used as controls and two independent lines of each genotype were used in the seedling growth assays. At least 25 seedlings of each genotype per treatment were used for the experiment.

Total RNA Extraction and Quantitative RT-PCR

Twelve-day-old seedlings of different genotypes were treated with BL (1 μM) or BRZ (2 μM) along with mock (MS) and ground to a fine powder with mortar and pestle in liquid nitrogen. Spectrum^{TM} Plant Total RNA kit (Sigma-Aldrich) was used to extract RNA following the manufacturer's recommendations. The RNA was quantified using NanoDrop^{TM} 1000 Spectrophotometer (Thermo Scientific) and about one microgram of total RNA was used for cDNA synthesis after DNase treatment with Promega DNase I. cDNA synthesis was undertaken with Invitrogen SuperScript-II Reverse Transcriptase following manufacturer's guidelines.

Quantitative real-time PCR was conducted using Brilliant III Ultra-Fast SYBR QPCR master mix (Agilent) in conjunction with Rotor-Gene Q (QIAGEN) and analysis was undertaken with the software provided using comparative quantification methods. *ACTIN7* (*At5g09810*) was used as the house-keeping gene for normalization. The experiments were repeated three times.

Immunoprecipitation and Co-immunoprecipitation Assay

Arabidopsis transgenic plants were used to extract total protein was isolated for IP using the extraction buffer containing 100mM Tris-HCl, pH 8.0, 0.1% [w/v] SDS, 0.5% [w/v] Sodium deoxycholate, 1% [v/v] glycerol, 50 mM sodium metabisulfite, 20 mM N-Ethylmaleimide (NEM) and protease inhibitor cocktail (Roche). Anti-GFP IP was performed. Total protein was incubated with 50 μL anti-GFP beads (Chromotek anti-GFP beads) and incubated on ice for 30 min. The beads were centrifuged down at 10,000 g for 1 min and washed three times with 1 mL of cold IP buffer. After the last wash 50 μL of pre-heated (95°C) 1 × SDS-loading buffer was used to elute the immuno-complex and analyzed on 10% SDS-PAGE using immunoblotting methods with Abcam (Cambridge, UK) anti-GFP and anti-SUMO1/2 antibodies generated against *At*SUMO1. The experiments were repeated at least three times.

N. benthamiana plants were infiltrated with respective constructs and total protein was isolated for co-IP using the extraction buffer containing 50 mM HEPES (pH7.5), 1 mM EDTA, 0.5% Trition-X 100, 1 mM DTT. Anti-GFP IP and anti-myc IP were performed. Total protein was incubated with 50 μL anti-GFP beads (Chromotek anti-GFP beads) and incubated on ice for 30 min. The beads were centrifuged down at 10,000 g for 1 min and washed three times with 1 mL of cold IP buffer. After the last wash 50 μL of pre-heated (95°C) 1 × SDS-loading buffer was used to elute the immuno-complex and analyzed on 10% SDS-PAGE using immunoblotting methods with Abcam (Cambridge, UK) anti-GFP and anti-HA antibodies. The experiments were repeated three times.

Protein Extraction and Western Blot Analysis

Frozen plant tissue was ground to a fine powder with a chilled pestle and mortar. Protein extraction buffer (50 mM Tris/HCl, pH 8.5, 4% SDS, 2% β-mercaptoethanol, 10 mM EDTA) and protease inhibitor tablet was added 1:1 w/vol. The mixture was centrifuged at 12000 g at 4°C for 10 min. The protein concentration was determined using a Direct Detect TM Infra-red Spectrometer (EMD Millipore) and samples were equalized with the addition of extraction buffer. Protein loading dye (4x) was added and the samples were separated on polyacrylamide gels. The proteins were transferred to a polyvinylidene difluoride (PVDF) membrane and blocked with 5% semi-skimmed milk powder at room temperature and probed with the respective antibodies. Secondary horseradish peroxidase (HRP)-conjugated antibodies were applied before developing the blots with X-ray film using an automated developer.

Confocal Microscopy, Quantification of Fluorescence Signal and Imaging

Confocal microscopy was performed by using a Zeiss 880 Airyscan system after propidium iodide staining. At least five roots were analyzed for each treatment in each experiment in three independent biological replicates. Four day old $_{pro}$*BZR1:BZR1-GFP* and $_{pro}$*BZR1::BZR1*$^{2K/R}$*-GFP* seedlings were used for the study. Identical laser settings were used within the same experiment. Nuclei/Cytosol (N/C) ratios for BZR1-YFP and BZR1$^{2K/R}$-GFP transgenics was calculated. For colocalization experiment, four-week-old *N. benthamiana* plants were infiltrated on the abaxial side of the leaf with infiltration media (10 mM $MgCl_2$ and 150 μg/ml acetosyringone) and *Agrobacterium tumefaciens* bacteria suspended in infiltration media. Agrobacterium cultures were prepared following a published protocol. Agrobacterium harboring expression constructs were infiltrated at an OD600 of 0.1 into *N. benthamiana* 72 h prior to confocal imaging. Sections of N. *benthamiana* leaves transiently expressing BZR1-GFP, BZR1$^{2K/R}$-GFP or GFP only and/or ULP1a-mCherry proteins were randomly sampled and mounted in water. Imaging was conducted with Zeiss LSM 880 laser scanning confocal microscope (LSCM) with Airyscan module. The excitation wavelength was 488 nm for GFP, and 594 nm for mCherry. Emission was detected using BP 495-550nm for GFP and LP 605nm filter for mCherry, airyscan processing was done using automatic Weiner filter settings.

QUANTIFICATION AND STATISTICAL ANALYSIS

All statistical analysis was performed using GraphPad Prism 6 software. One-way or Two-way ANOVAs with post hoc Turkey test were performed at a significance level of $p < 0.05$ or $p < 0.01$ or $p < 0.001$. All root phenotype experiments had at least an n = 25-30 seedlings in each biological replication. Data are representing an average of three individual biological replicates.

DATA AND CODE AVAILABILITY

The published article includes all datasets generated or analyzed during this study.

Current Biology, Volume *30*

Supplemental Information

SUMO Conjugation to BZR1 Enables Brassinosteroid Signaling to Integrate Environmental Cues to Shape Plant Growth

Moumita Srivastava, Anjil K. Srivastava, Beatriz Orosa-Puente, Alberto Campanaro, Cunjin Zhang, and Ari Sadanandom

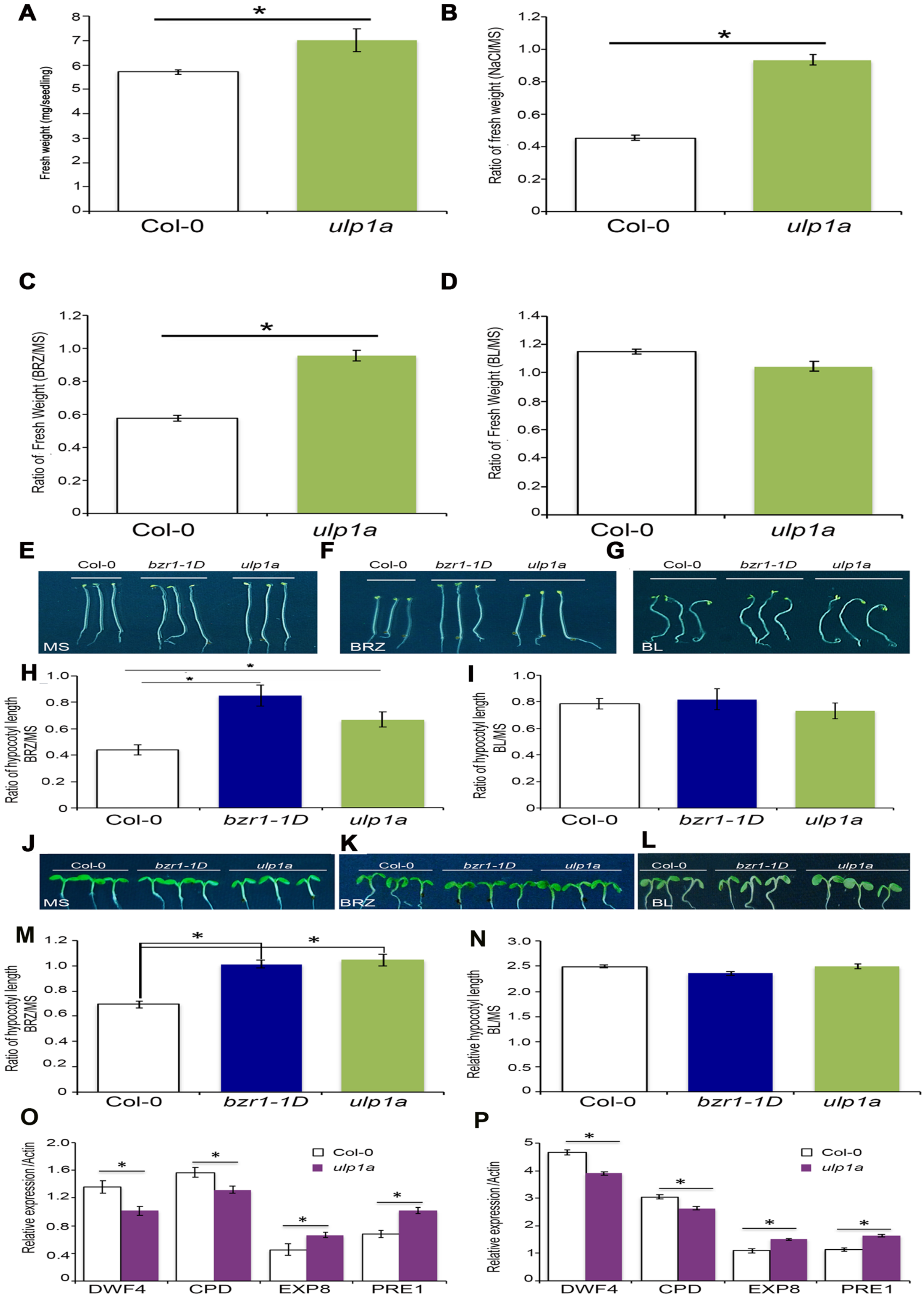

Figure S1; ULP1a is the SUMO protease involved in BR signaling. Related to Figure 1. (A) Quantification for fresh weight for Col-0 and *ulp1a* grown on MS for 12 days. (B-D) Quantification for relative fresh weight for 12d old Col-0 and *ulp1a* grown on BRZ (2μM) (B), BL(1μM) (C) and NaCl (100mM) with reference to untreated samples. *ulp1a* is less sensitive to BRZ mediated inhibition of seedling growth. (E-G) Representative image of 6d old seedlings of Col-0, *bzr1-1D* and *ulp1a* grown on ½ MS, BRZ (2μM) and BL(1μM) medium respectively in dark. (H-I). Quantification of relative root growth for 6d old seedlings treated with BRZ (2μM) (H) and BL(1μM) (I) in reference to untreated seedlings. (J-L). Representative image of 6d old seedlings of Col-0, *bzr1-1D* and *ulp1a* grown on ½ MS, BRZ (2μM) and BL(1μM) medium respectively in light. (M-N). Quantification of relative root growth for 6d old seedlings treated with BRZ (2μM) (M) and BL(1μM) (N) in reference to untreated seedlings. (O-P) qRT-PCR analysis for Brassinosteroid target genes in 10d old Col-0 and ulp1a seedlings in untreated (O) and BRZ treated (P) conditions.

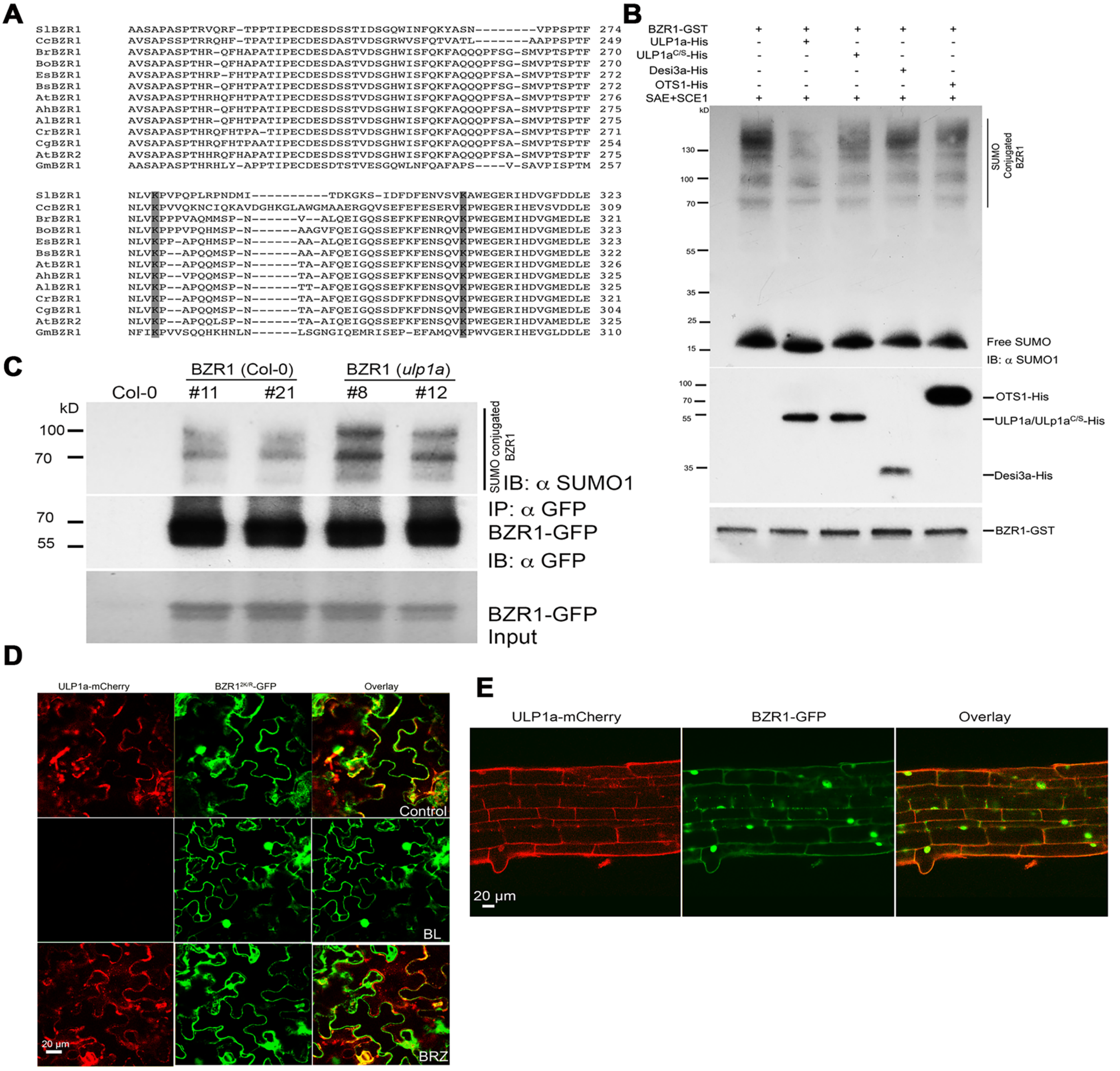

Figure S2, SUMO sites for BZR1 are conserved in different plant species and is deSUMOylated by the SUMO protease ULP1a. Related to Figure 2; (A) Amino acid alignment of BZR1 from Arabidopsis thaliana with *Solanum lycopersicum* (*Sl*BZR1), *Cajanus cajan* (CcBZR1), *Brassica rapa* (*Br*BZR1), Brassica olaracea (*Bo*BZR1), *Eutrema salsugineum* (*Es*BZR1), Boechera stricta (BsBZR1), Arabidopsis halleri (*Ah*BZR1), *Arabidopsis lyrata* (*Al*BZR1), *Capsella rubella* (*Cr*BZR1), Capsella grandiflora (*Cg*BZR1), *Arabidopsis thaliana* (*At*BZR2/BES1) and *Glycine max* (*Gm*BZR1). The conserved lysine at the two positions are highlighted in grey. Phytozome (http://phytozome.jgi.doe.gov/pz/portal.html) was used to obtain the BZR1 homolog sequences for different plant species. (B) ULP1a specifically deSUMOylates BZR1. High molecular weight conjugates of His-SUMO1 modified GST tagged BZR1 were formed by incubating purified SUMO E1 (SAE1 and 2) and E2 (SCE1) with BZR1-GST in the presence His-ULP1a, His-ULP1a$^{C/S}$, His-OTS1 and His-Desi3a and subsequently immunoblotted with anti-SUMO1 (αSUMO1) (upper panel) to detect SUMO chains, anti-His (αHis) (middle panel) to detect His-ULP1a, His-ULP1a$^{C/S}$, His-OTS1 and His-Desi3a and anti-GST (α GST) to detect BZR1-GST (lower panel). (C) BZR1 gets hyperSUMOylated in *ulp1a* background. Immunoprecipitation (IP: αGFP) experiments were carried out with anti-GFP beads from total protein derived from transgenic lines expressing $_{pro}$BZR1::BZR1-GFP expressed in Col-0 or $_{pro}$BZR1::BZR1-GFP expressed in ulp1a background. Immunoblots were probed with anti-GFP (IB: αGFP) or anti- SUMO1/2 (IB: αSUMO1) antibodies. (D) ULP1a colocalises with BZR1$^{2K/R}$ in the cytoplasm. *N. benthamiana* leaves co-infiltrated with ULP1a-mCherry and BZR1$^{2K/R}$-GFP were analyzed for fluorescence after 3 days. Before imaging, the leaves were infiltrated with 1µM BL or 2µM BRZ and incubated for 1 hour to see the effect of the treatments. Images were obtained using confocal laser scanning microscope Carl Zeiss Airyscan 880. Scale bar= 20µm. (E) BZR1 and ULP1a co-localise in stable transgenic lines in Arabidopsis. Confocal images of transgenic Arabidopsis seedlings co-expressing $_{pro}$BZR1::BZR1-GFP and 35S:ULP1a:mcherry.

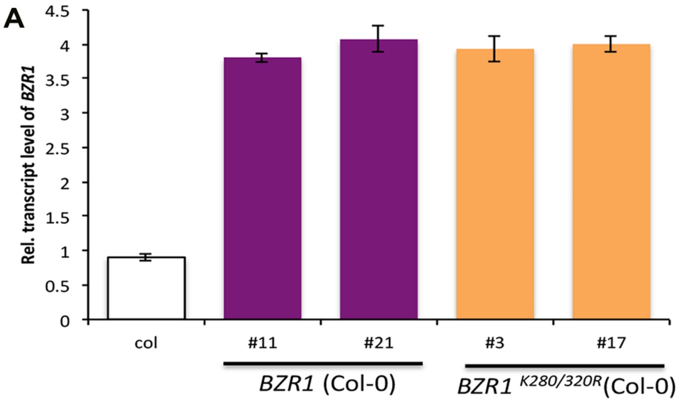

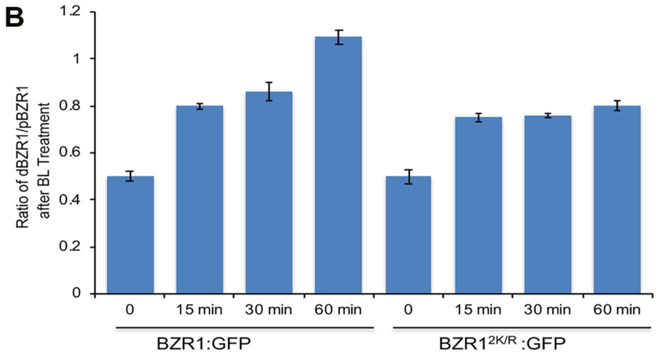

Figure S3, $_{pro}$BZR1::BZR1-GFP and $_{pro}$BZR1::BZR1$^{2K/R}$-GFP have similar transcript levels but respond differently in response to the brassinosteroids. Related to Figure 3. (A) Expression analysis of BZR1 in different transgenic lines. The expression levels of BZR1 in Col-0 and different transgenic plants by real-time PCR analysis. The experiment was replicated three times and data shown mean ± SD (N=3). (B) BL treatment does not affect the ratio of phosphorylated and dephosphorylated forms of BZR1 in $_{pro}$BZR1::BZR1$^{2K/R}$-GFP. Ratio of the band intensities for dephosphorylated BZR1 to phosphorylated BZR1 for BZR1 (Col-0) and BZR1$^{2K/R}$ (Col-0) when treated with BL at different time points (Figure 3E) quantified using image J software.

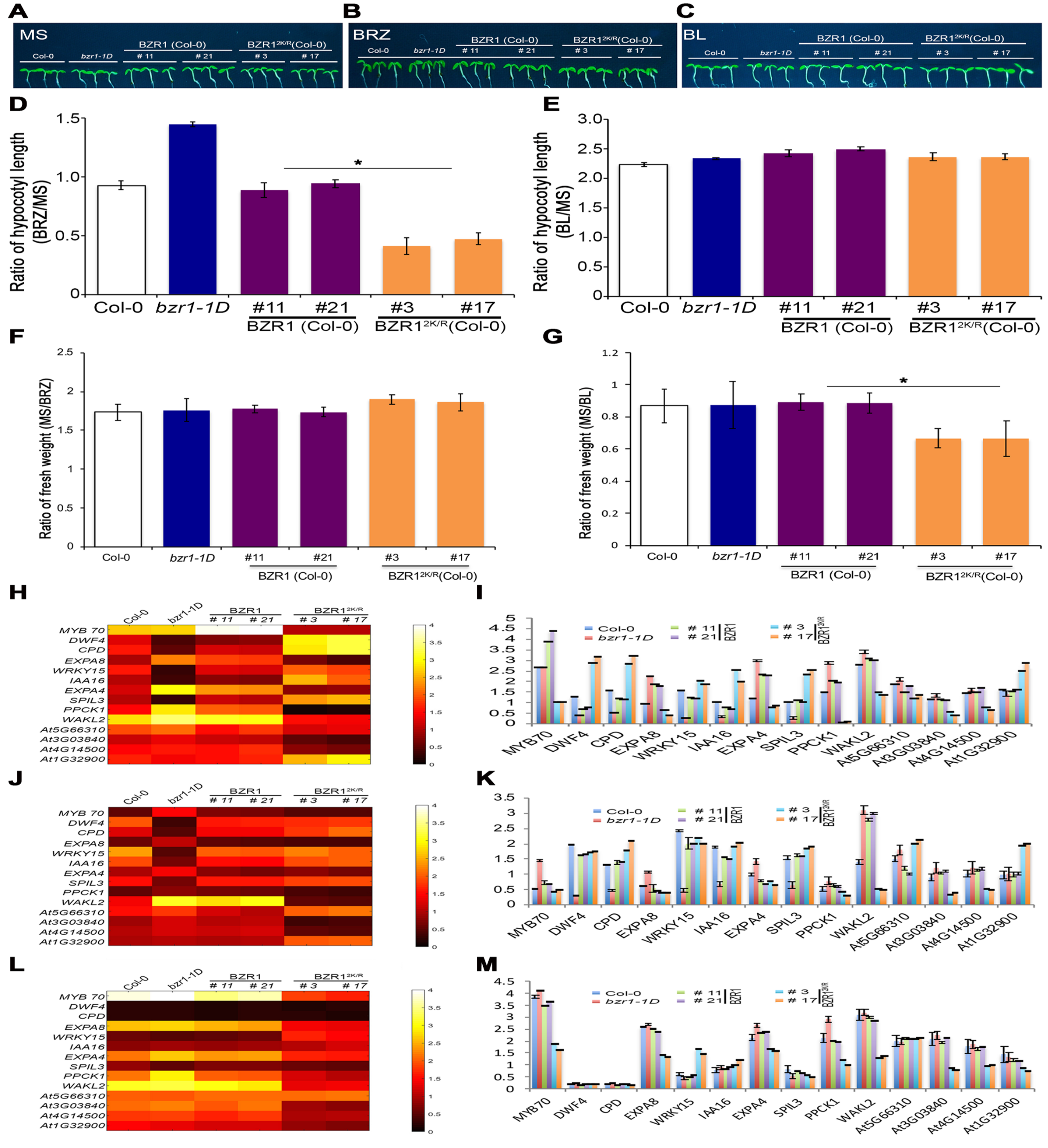

Figure S4; SUMO deficient form of BZR1 is hypersensitive to BR deficiency. Related to Figure 4. A-C. A-C Representative images of hypocotyl lengths of 6d old seedlings of Col-0, *bzr1-1D*, $_{pro}$BZR1::BZR1-GFP, $_{pro}$BZR1::BZR1$^{2K/R}$-GFP grown on ½ MS, BRZ (2μM) and BL (1μM) medium respectively in light. D-E. Relative growth of hypocotyls for seedlings grown in BRZ (D) or BL (E) with reference to the untreated seedlings. F-G Relative fresh weight for 10d old seedlings of Col-0, *bzr1-1D*, $_{pro}$BZR1::BZR1-GFP and $_{pro}$BZR1::BZR1$^{2K/R}$-GFP grown on BRZ (2μM) (F) and BL (1μM) (G) medium with reference to untreated seedlings. H-M. SUMO mutant form of BZR1 fails to function as a transcription factor. Heat map and quantification of the BL target genes for which the expression was studied in Col-0, bzr1-1D, transgenic $_{pro}$BZR1::BZR1-GFP and transgenic $_{pro}$BZR1::BZR1$^{2K/R}$-GFP transgenic lines without any treatment (H and I), on treatment with 2μM BRZ (J and K) and treated with 1μM BL (L and M). RNA was isolated from10 day old seedlings treated with respective treatments for 5 hours and used for cDNA preparation. Real Time PCR analysis was done which confirmed a defective transcription factor activity for the SUMO deficient form of BZR1.

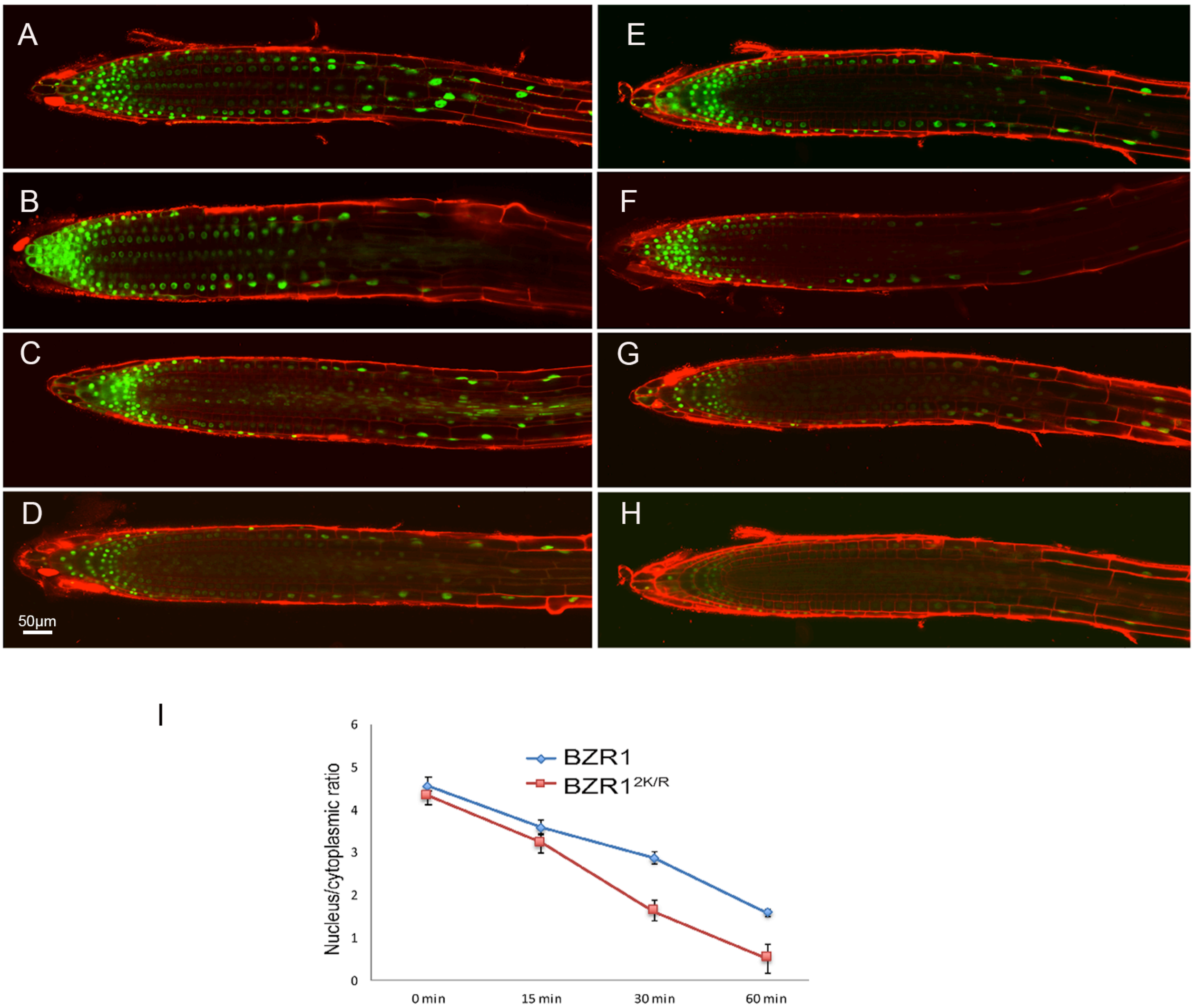

Figure S5; BRZ causes rapid degradation of $_{pro}$BZR1:BZR12K/R-GFP. Related to Figure 5. A-D. Confocal images of 4d old proBZR1::BZR1-GFP seedlings at 0, 15, 30 and 60 minutes of treatment with 2µM BRZ respectively. E-H. Confocal images of 4d old $_{pro}$BZR1::BZR1$^{2K/R}$-GFP seedlings at 0, 15, 30 and 60 minutes of treatment with 2µM BRZ respectively. I N/C ratios of the GFP signals from the elongation zone of the roots at 0, 15, 30 and 60 min time points after treatment with 2µM BRZ.

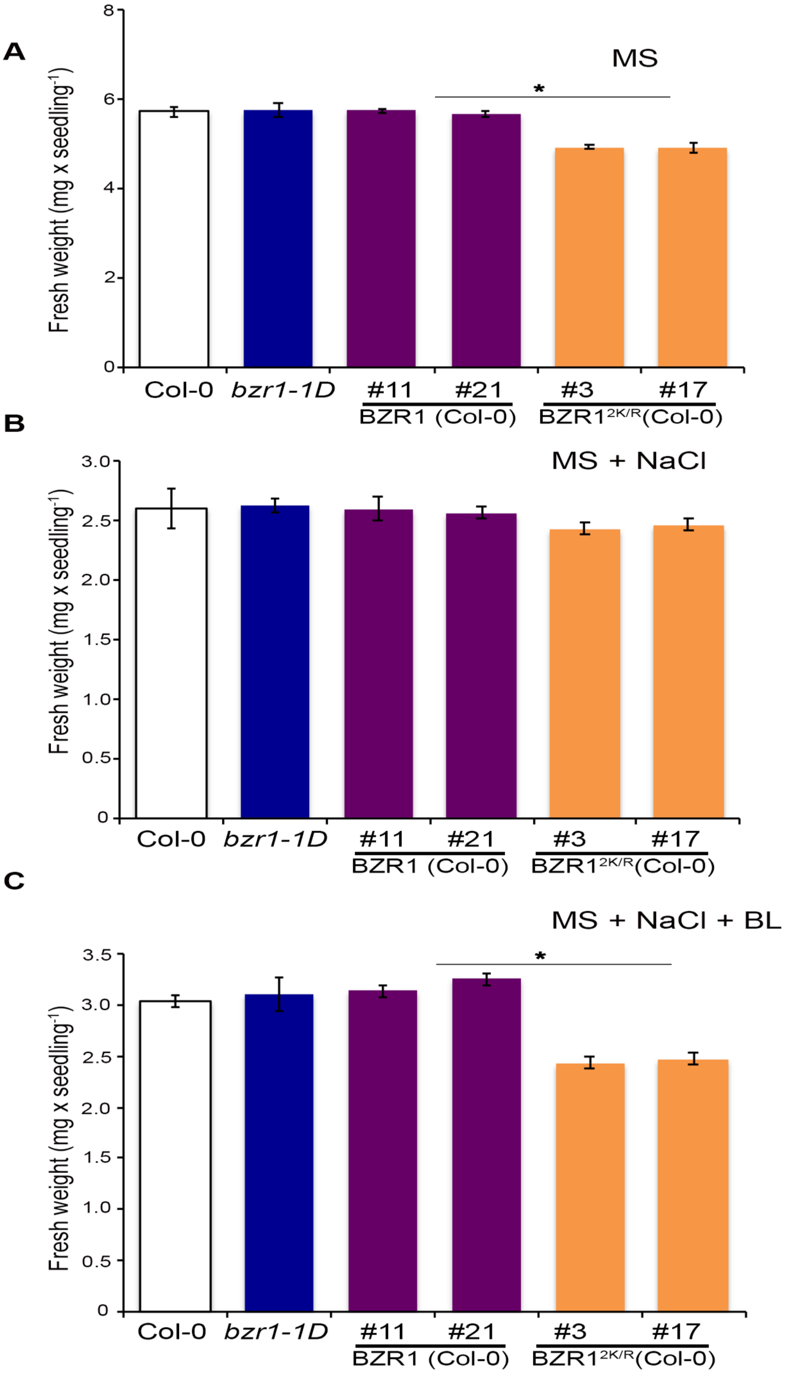

Figure S6; Brassinosteroids cannot rescue the short root phenotype for proBZR1::BZR1$^{2K/R}$-GFP seedlings. Related to Figure 7. (A) Quantification of fresh weight measurements for 12d old Col-0, *bzr1-1D*, $_{pro}$BZR1::BZR1-GFP and $_{pro}$BZR1::BZR1$^{2K/R}$-GFP plants grown on MS. (B) Quantification of fresh weight measurements of 12d old Col-0, *bzr1-1D*, $_{pro}$BZR1::BZR1-GFP and $_{pro}$BZR1::BZR1$^{2K/R}$-GFP plants grown on 100mM NaCl. (C) Quantification of fresh weight measurements of 12d old Col-0, *bzr1-1D,* $_{pro}$BZR1::BZR1-GFP and $_{pro}$BZR1::BZR1$^{2K/R}$-GFP plants grown on 100mM NaCl for 6 days and then on 1µM BL for next 6 days.

Genes	Forward primer	Reverse primer	Function
proBZR1	5'CACCGGTCTCAAGTAGCCTA ATTCATCGAACCCTCT 3'	5'TTTGGTCTCATCATCGGGAA AACCAACAACCAA 3'	Cloning
BZR1	5' CACCATGACTTCGGATGGAGC TACG 3'	5' ACCACGAGCCTTCCCATTTC 3'	Cloning
proBZR1::BZR1 overlap	5' CGTAGCTCCATCCGAAGTCAT CGGGAAAACCAACAACCAA 3'	5' TTGGTTGTTGGTTTTCCCGAT GACTTCGGATGGAGCTACG 3'	Cloning
BZR1K280R	5' TCAATCTTGTGAGACCTGCGC T 3'	5' AGGCGCAGGTCTCACAAGATT GA 3'	Site-directed mutagenesis
BZR1K320R	5' TCAAAGCTCTCAGTTTAAATTT GAGAATAG 3'	5' CTATTCTCAAATTTAAACTGAG AGCTTTG 3'	Site-directed mutagenesis
BZR1	5' GTTACCCCACCGGTCTCATC 3'	5' TGAAACTGGTGGCGATGTGT 3'	Genotyping
BIN2	CACCATGGCTGATGATAAGGA GATG	AGTTCCAGATTGATTCAAGAA GCTT	Cloning
ULP1a	5' CACCATGAAAAACCAATCTAG GG 3'	5' CTCGGCTTTCAGTTGCAGA 3'	Cloning
SAUR8	5' GGGACACTTCCCTGTCTACG 3'	5' GGAATGGTGAGACCCATGTCA 3'	Q-PCR
ulp1a	5′ GCCTTTTCAGAAATGGATAAA TAGCCTTGCTTCC 3′	5' CTCGGCTTTCAGTTGCAGA 3'	genotyping
SCE1	5' CACCATGGCTAGTG GAATC 3'	5' TTA GACAAGAGCA GGATAC 3'	Cloning
Actin	5' CCATCGCTCATCGGAATGGA 3'	5' TGGAACCACCACTGAGAACG 3'	Q-PCR
ULP1a	5' TGGTTACCACAAACGGAGCG 3'	5' TGAACTTGTCTCCGCAACGA 3'	Genotyping
MYB70	5' GGCGGAGGAAGATGATACGA 3'	5' ACAACTCTGCCCTTCCTCTC 3'	Q-PCR
DWF4	5' GGTGGAAAGTGTTACCGGTG 3'	5' TCCTCCAAACGGCATGTAGT 3'	Q-PCR
CPD	5' TCTCCCTCTCTTCTCCACCA 3'	5' GCGACAAGTAAAGCCACCAA 3'	Q-PCR
EXPA8	5'GTTTTACGGCGGCGAAGATG 3'	5'CCACCTCGGGTCATCGTTAC 3'	Q-PCR
WRKY15	5'GGCGGAGGAAGATGATACG A 3'	5' ACAACTCTGCCCTTCCTCTC3'	Q-PCR
IAA16	5'	5'	Q-PCR

	TGCTTGTAGGAGACGTACCG3’	CAACCGATCGAAACAGGCTT3’	
EXPA4	5’ AACTCCCTGAATCAGCCACA3’	5’ CCTTCGCCTTATCCGCAAAA3’	Q-PCR
SPIL3	5’ TGCCTCCTCCATGCATGTTA3’	5’ TGGGCTTATACGGGCTTTGA3’	Q-PCR
PPCK1	5’ AGGAGTGTCGGGCATGTAAA3’	5’ CATCGCCAAACTCAGAAGCA3’	Q-PCR
WAKL2	5’ AAGCTCCCGTAACTTCCTCC3’	5’ ATACCCGCCATAACAGCCTT3’	Q-PCR
At5G66310	5’ GGCCATCTGATGCTTCTGTG3’	5’ TTGCTGCGTCCTTGAAACTC3’	Q-PCR
At3G03840	5’ TTGCAGTGTACGTAGGGGAG3 ’	5’ TCTTCAGGACAAGGGATCGT3’	Q-PCR
At4G14500	5’ GGCCTTCACTCTGGGATCAT3’	5’ AACCACATGCCCTTCTCAGA3’	Q-PCR
At1G32900	5’ GCTGGTAAGATTGTGTGTGAG	5’ CACAACACAAGTGTCCCAAG	Q-PCR

Table S1: List of primers used in the study, Related to STAR methods

Genes	Constructs	Vectors	Experiment
BZR1	BZR1:GFP	pEG103	Co-IP/Confocal
BZR1$^{2K/R}$	BZR1$^{2K/R}$:GFP	pEG103	Co-IP/Confocal
proBZR1::BZR1	BZR1:GFP	pMDC107	Generation of transgenics/Confocal
proBZR1::BZR1$^{2K/R}$	BZR1$^{2K/R}$:GFP	pMDC107	Generation of transgenics/Confocal
BZR1	GST:BZR1	pDEST15	In-vitro pull down assay
BIN2	HA:BIN2	pEG201	Co-IP
BIN2	His:BIN2	pDEST17	In-vitro pull down assay
ULP1a	HA:ULP1a	pEG201	Co-IP
ULP1a	ULP1a:mcherry	pMDC43	Confocal
ULP1a	His:ULP1a	pDEST17	In-vitro pull down assay
ULP1a	HA:ULP1a	pGWB15	Generation of transgenics
SCE1(E2)	HA:SCE1	pEG201	Co-IP
SUMO1	His:SUMO1	pDEST17	Isopeptide cleaved SUMO chains

Table S2: List of constructs used in the study, Related to STAR Methods
